# Supplementary material for: Discovery of the Oncogenic Parp1, a Target of bcr-abl and a Potential Therapeutic, in mir-181a/PPFIA1 Signaling Pathway
Source: Mol Ther Nucleic Acids. 2019 Feb 8;16:1–14. doi: 10.1016/j.omtn.2019.01.015 (PMC6393709; doi:10.1016/j.omtn.2019.01.015)
Supplement: Document S1. Figures S1–S4, Tables S1–S5, and Supplemental Materials and Methods [file mmc1.pdf]

OMTN, Volume *16*

Supplemental Information

Discovery of the Oncogenic Parp1, a Target of bcr-abl and a Potential Therapeutic, in mir-181a/PPFIA1 Signaling Pathway

Chunming Gu, Yanjun Liu, Zhao Yin, Juhua Yang, Guiping Huang, Xuejiao Zhu, Yumin Li, and Jia Fei

Supplemental Information

Supplemental Figures and Legends

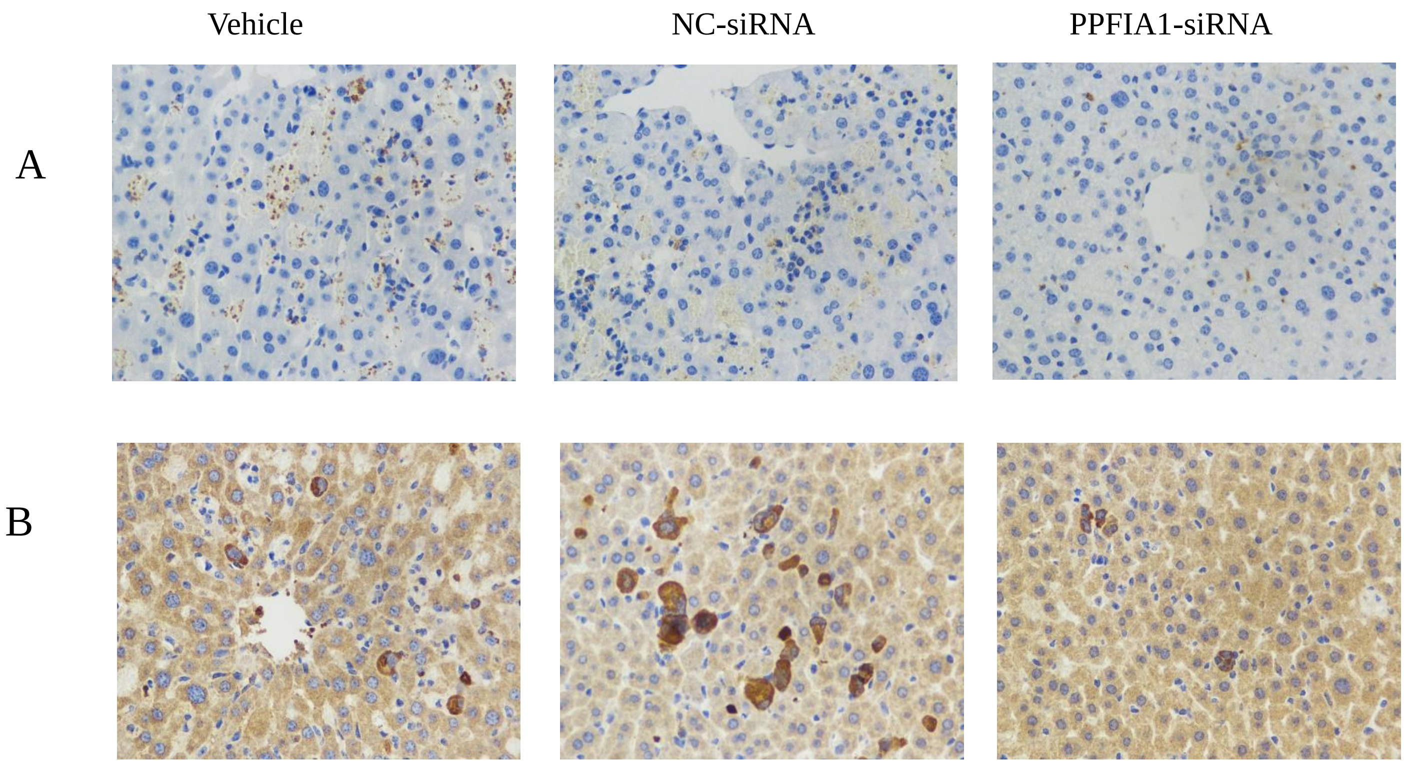

Figure S1. Immunohistochemical (IHC) analysis of liver samples from mice engrafted with K562-Luciferase cells untreated, NC-treated, and PPFIA1-siRNA-treated using anti-PPFIA1 (A) and anti-PARP1 (B) antibodies. Untreated and NC-treated mice were also used as controls.

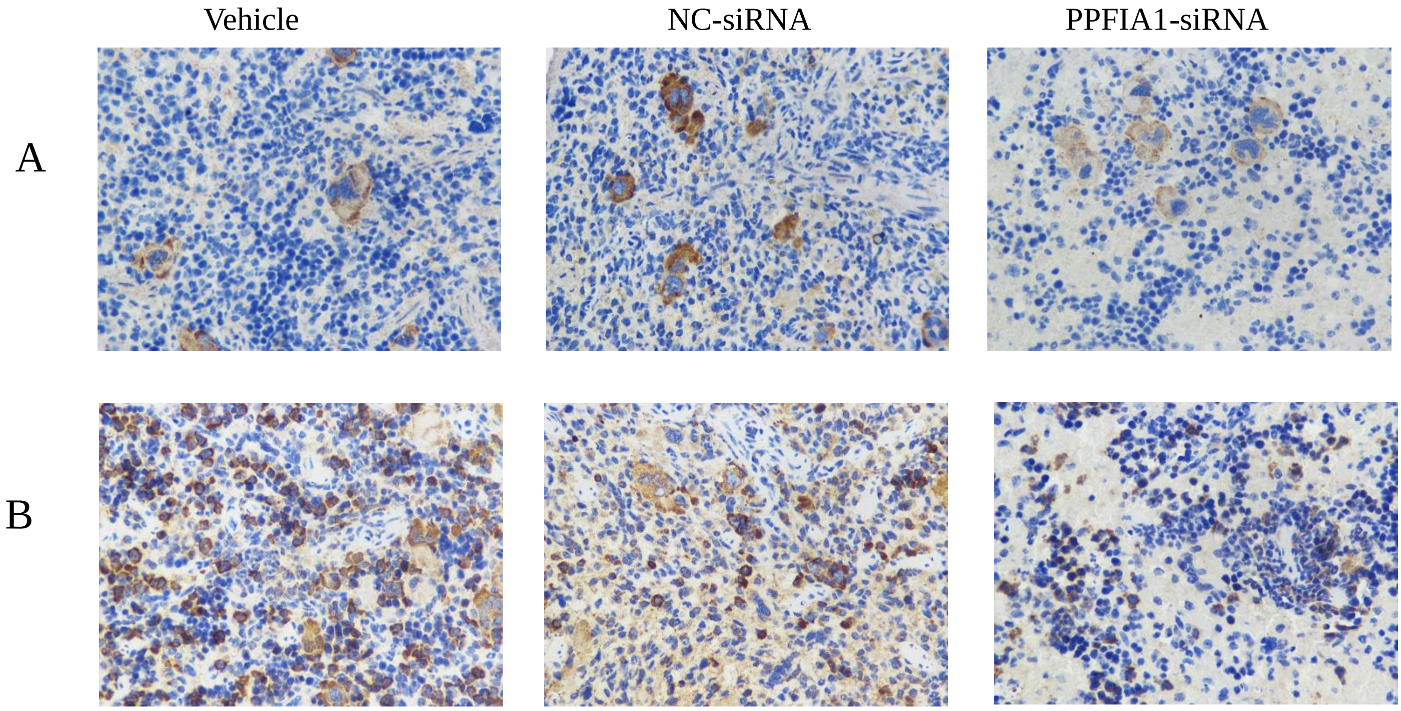

Figure S2. Immunohistochemical (IHC) analysis of spleens from mice engrafted with K562-Luciferase cells untreated, NC-treated, and PPFIA1-siRNA-treated using anti-PPFIA1 (A) and anti-PARP1 (B) antibodies. Untreated and NC-treated mice were also used as controls.

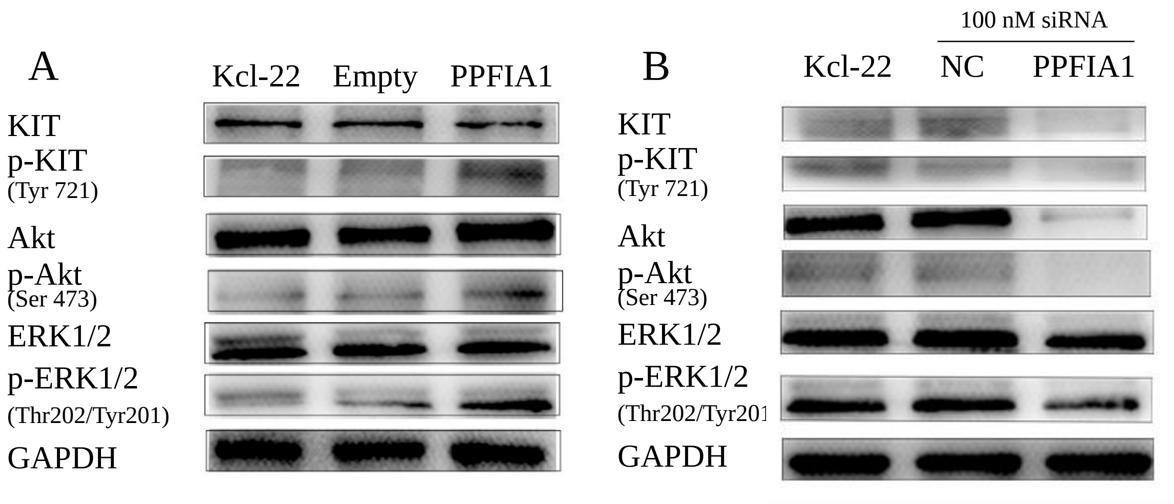

Figure S3. The effect of PPFIA1 on KIT signaling pathway.

(A) Overexpression of PPFIA1 increased the expression of phosphotylated KIT, Akt and ERK1/2 proteins in Kcl-22 cells. (B) Transfection of PPFIA1-siRNA for 48h attenuated the expression of phosphotylated KIT, Akt and ERK1/2 proteins in Kcl-22 cells.

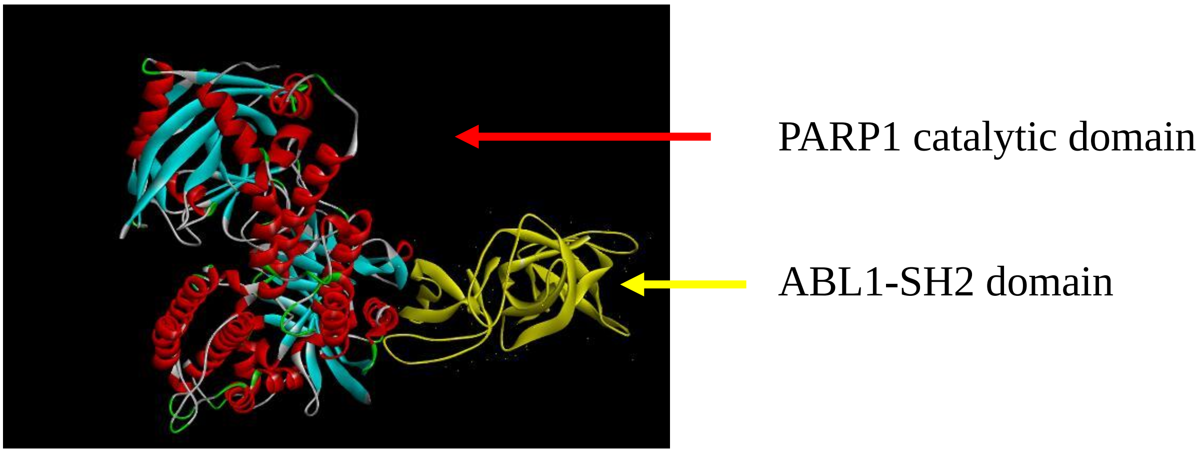

Figure S4. Docking of the PARP1 catalytic domain and the ABL1-SH2 domain.

Supplemental Tables

Table S1. Antibodies

REAGENT or RESOURCE	Supplier	IDENTIFIER
Antibodies		
Anti-GAPDH	Cell Signaling Technology	Cat# sc-74470
Anti-PPFIA1	Abcam	Cat# ab-96377
Anti-PPFIA1	Santa Cruz Biotechnology	Cat# sc-398030
Anti-C-KIT	Santa Cruz Biotechnology	Cat# sc-17806
Anti-p-C-KIT (Tyr 721)	Santa Cruz Biotechnology	Cat# sc-101659
Anti-PARP1	Santa Cruz Biotechnology	Cat# sc-74470
Anti-PARP	Cell Signaling Technology	Cat# 9532S
Anti-BCR	Abcam	Cat# ab-86173
Anti-c-Abl	Santa Cruz Biotechnology	Cat# sc-23
Anti-AKT	Cell Signaling Technology	Cat# 4691S
Anti-p-AKT (Ser 473)	Cell Signaling Technology	Cat# 4060S
Anti-ERK1/2	Cell Signaling Technology	Cat# 4695S
Anti-p-ERK1/2 (Thr 202/Thr 201)	Cell Signaling Technology	Cat# 9101S
Anti-rabbit IgG, HRP-linked	Cell Signaling Technology	Cat# 7074S
Anti-rabbit IgG, HRP-linked	Cell Signaling Technology	Cat# 7076S
Anti-P65	Santa Cruz Biotechnology	Cat# sc-514451
Anti-p-P65 (Ser 536)	Santa Cruz Biotechnology	Cat# sc-136548
Anti-FLAG	Sigma Aldrich	Cat# F1804

Table S2. Cell Lines

Cell lines	SOURCE
K562	Institute of Shanghai cell biology
293T	
KCL-22	Laboratory of Professor Markus Muschen
EM2	
Jurl-MK1	
B-luciferase K562	Beijing Biocytogen Co., Ltd.

Table S3. Experimental models

Experimental Model	SOURCE
Balb/c nu: female	Institute of laboratory animal science, JINAN University
C57BL/6	
B-NDG®(B-NSGTM) mice	Beijing Biocytogen Co., Ltd.

Table S4. Chemicals, recombinant proteins, and plasmids

Chemicals, Recombinant proteins, Plasmids	SOURCE	IDENTIFIER
Imatinib Mesylate (STI571)	Selleck.cn	S1026
Olaparib	Selleck.cn	S1060
PARP1 protein	Sino biological	11040-H08B-20
Anti-Flag Affinity Gel	Bimake	B23101
Psi-CHECK-2	Promega	C8021
PSI-CHECK-PPFIA1-3′UTR	This paper	N/A
PSI-CHECK-PPFIA1-MUT-3′UTR	This paper	N/A
LP-X0107-lv201-C0010	GeneCopoeia	N/A
LP-NEG-lv201-C0010	GeneCopoeia	N/A
LPP-X0107-Lv181-400	GeneCopoeia	N/A
LPP-NEG-Lv181-400	GeneCopoeia	N/A
EX-Z8307-Lv202	GeneCopoeia	N/A
EX-NEG-Lv202	GeneCopoeia	N/A
EX-Z4808-Lv201	GeneCopoeia	N/A
EX-NEG-Lv201	GeneCopoeia	N/A

Table S5. Oligonucleotides

Oligonucleotide	Sequence (5′ to 3′)	SOURCE
miR-181a mimic		Guangzhou RiboBio Co., Ltd.
sense	AACAUUCAACGCUGUCGGUGAGU	
antisense	UCACCGACAGCGUUGAAUGUUGU	
PPFIA1-siRNA		Guangzhou RiboBio Co., Ltd.
Sense	CCACAAAGCUCUGGAUGAAdTdT	
Antisense	dTdTGGUGUUUCGAGACCUACUU	
Scramble duplex, SCR		Guangzhou RiboBio Co., Ltd.
Sense	UUCUCCGAACGUGUCACGUTT	
Antisense	ACGUGACACGUUCGGAGAATT	
PPFIA1 primers		Sangon Biotech (Shanghai) Co., Ltd.
Forward	GCTTVAGTTAACCTTTTTCATGTATATCCA	
Reverse	TCGACAGTTAACATAAACGTAAAATCCTT	
GAPDH Primers		Sangon Biotech (Shanghai) Co., Ltd.
Forward	CAACGGATTTGGTCGTATT	
Reverse	CACAGTCTTCTGGGTGGC	
KIT Primers		Sangon Biotech (Shanghai) Co., Ltd.
Forward	CAATGGCACGGTTGAATGTA	
Reverse	AAGGAGTGAACAGGGTGTGG	
PARP1 Primers		Sangon Biotech (Shanghai) Co., Ltd.
Forward	CCGCATACTCCATCCTCAGT	
Reverse	GCTTCTTCATCCCAAAGTCG	
P65 Primers		Sangon Biotech (Shanghai) Co., Ltd.

Forward	GGAGCACAGATACCACCAAGA
Reverse	CGGCAGTCCTTTCCTACAAG

Supplemental Methods

Immunohistochemical staining and image analysis

The slides were deparaffinised and rehydrated following the manufacturer's instructions. Endogenous peroxidase activity was abolished by 3% H_2O_2, antigen retrieval was conducted in citrate buffer. Slices were incubated with a primary antibody (anti-PARP1 or PPFIA1 at 1:50 dilution) at 4 ℃ overnight, followed by incubation with a HRP-labeled secondary antibody for 30 min. Images were obtained by a Leica DMRI microscope installed on a Lexica DC500 camera (Leica).
