# Supplementary material for: Discovery of the Oncogenic Parp1, a Target of bcr-abl and a Potential Therapeutic, in mir-181a/PPFIA1 Signaling Pathway
Source: Mol Ther Nucleic Acids. 2019 Feb 8;16:1–14. doi: 10.1016/j.omtn.2019.01.015 (PMC6393709; doi:10.1016/j.omtn.2019.01.015)
Supplement: Document S2. Article plus Supplemental Information [file mmc8.pdf]

Molecular Therapy
Nucleic Acids
Original Article

Discovery of the Oncogenic Parp1, a Target of bcr-abl and a Potential Therapeutic, in mir-181a/PPFIA1 Signaling Pathway

Chunming Gu,[1,2,3,4,5] Yanjun Liu,[1,3,4,5] Zhao Yin,[1,2,3,4,5] Juhua Yang,[1,3,4] Guiping Huang,[1,3,4] Xuejiao Zhu,[1,3,4] Yumin Li,[1,3,4] and Jia Fei[1,2,3,4]

[1]Department of Biochemistry and Molecular Biology, Medical College of Jinan University, Guangzhou 510632, China; [2]Insititute of Chinese Integrative Medicine, Medical College of Jinan University, Guangzhou 510632, China; [3]Engineering Technology Research Center of Drug Development for Small Nucleic Acid, Guangdong, China; [4]Antisense Biopharmaceutical Technology Co., Ltd., Guangzhou, China

miR-181a is downregulated in leukemia and affects its progression, drug resistance, and prognosis. However, the exact mechanism of its targets in leukemia, particularly in chronic myelogenous leukemia (CML), has not previously been established. Here, we use a multi-omics approach to demonstrate that protein tyrosine phosphatase, receptor type, f polypeptide, leukocyte common antigen (LAR) interacting protein (liprin), alpha 1 (PPFIA1) is a direct target for miR-181a in CML. Phosphoarray assay shows that multiple phosphorylated proteins, particularly KIT signaling molecules, were downregulated in PPFIA1 inhibition. Additionally, PPFIA1 bound PARP1, a common molecule downstream of both PPFIA1 and BCR/ABL, to upregulate KIT protein through activation of nuclear factor kappa B (NF-κB)-P65 expression. Targeted inhibition of PPFIA1 and PARP1 downregulated c-KIT level, inhibited CML cell growth, and prolonged mouse survival. Overall, we report a critical regulatory miR-181a/PPFIA1/PARP1/NF-κB-P65/KIT axis in CML, and our preclinical study supports that targeted PPFIA1 and PARP1 may serve as a potential CML therapy.

INTRODUCTION

Chronic myelogenous leukemia (CML) is a clonal myeloproliferative disorder with approximately 1–2 people per 100,000 population, and accounts for 15% of all leukemia in adults.[1] The Philadelphia chromosome (Ph) is a specific genetic abnormality with a t(9,22) reciprocal chromosomal translocation in leukemia cells (particularly CML cells). This translocation leads to expression of the BCR/ABL fusion protein, which is a constitutively active tyrosine kinase.[2] The tyrosine kinase inhibitor, imatinib (IM; Gleevec; Novartis Pharma, Basel, Switzerland), potently inhibits the tyrosine kinase activities of BCR/ABL, as well as the stem cell factor receptor, KIT. IM shows remarkable therapeutic effect as a component of frontline treatment regimens for CML.[3] The capacity of IM to inhibit non-BCR/ABL1 targets has expanded its use to treatment of malignancies driven by KIT mutations, which has been implicated in CML pathogenesis. Dual inhibition of BCR/ABL and KIT is required for suppression of mature CML progenitors, and the stem cell factor (SCF)/KIT pathway must be inhibited to enable apoptosis induced by BCR/ABL inhibitors in CML cells.[4,5]

MicroRNAs (miRNAs) have recently been implicated in the regulation of a number of biological processes.[6,7] The miR-181 family of miRNA precursors includes four members (miR-181a, miR-181b, miR-181c, and miR-181d) and is dysregulated in hematopoiesis and hematological malignancies. miR-181 is conserved in vertebrates, and its ectopic expression in hematopoietic and progenitor cells promotes blood cell development and differentiation.[8] Lin et al.[9] performed a meta-analysis to determine the prognostic role of miR-181a/b in human cancers. Analysis of 11 studies, including 1,252 patients, demonstrated that high miR-181a/b expression can prolong overall survival (OS) in patients with hematological malignancies, compared with low expression (hazard ratio [HR] = 0.717, p = 0.0001). miR-181 is differentially expressed in a variety of types of leukemia. The downregulation of miR-181a as a tumor suppressor has been reported in aggressive chronic lymphocytic leukemia (CLL), CML, and acute myeloid leukemia (AML).[10–12] However, the exact mechanism of miR-181a targets in leukemia, particularly CML, is poorly understood.

An important step toward determining the gene-regulatory activity of miRNAs is accurate prediction of their targets and monitoring of their expression levels. Several computational target prediction tools have been developed, including TargetScan, miRFocus, and miRvestigator;[13] however, these *in silico* target prediction tools suffer from high false-positive rates, because they use only sequence complementarity and assume structural stability (following putative assembly) to predict specific targets of miRNA.[14] To identify the

Received 1 April 2018; accepted 30 January 2019;
https://doi.org/10.1016/j.omtn.2019.01.015.

[5]These authors contributed equally to this work.

Correspondence: Jia Fei, Department of Biochemistry and Molecular Biology, Medical College of Jinan University, 601 Western Huangpu Avenue, 510632 Guangzhou, China.
E-mail: efeijia@163.com

This is an open access article under the CC BY license (http://creativecommons.org/licenses/by/4.0/).

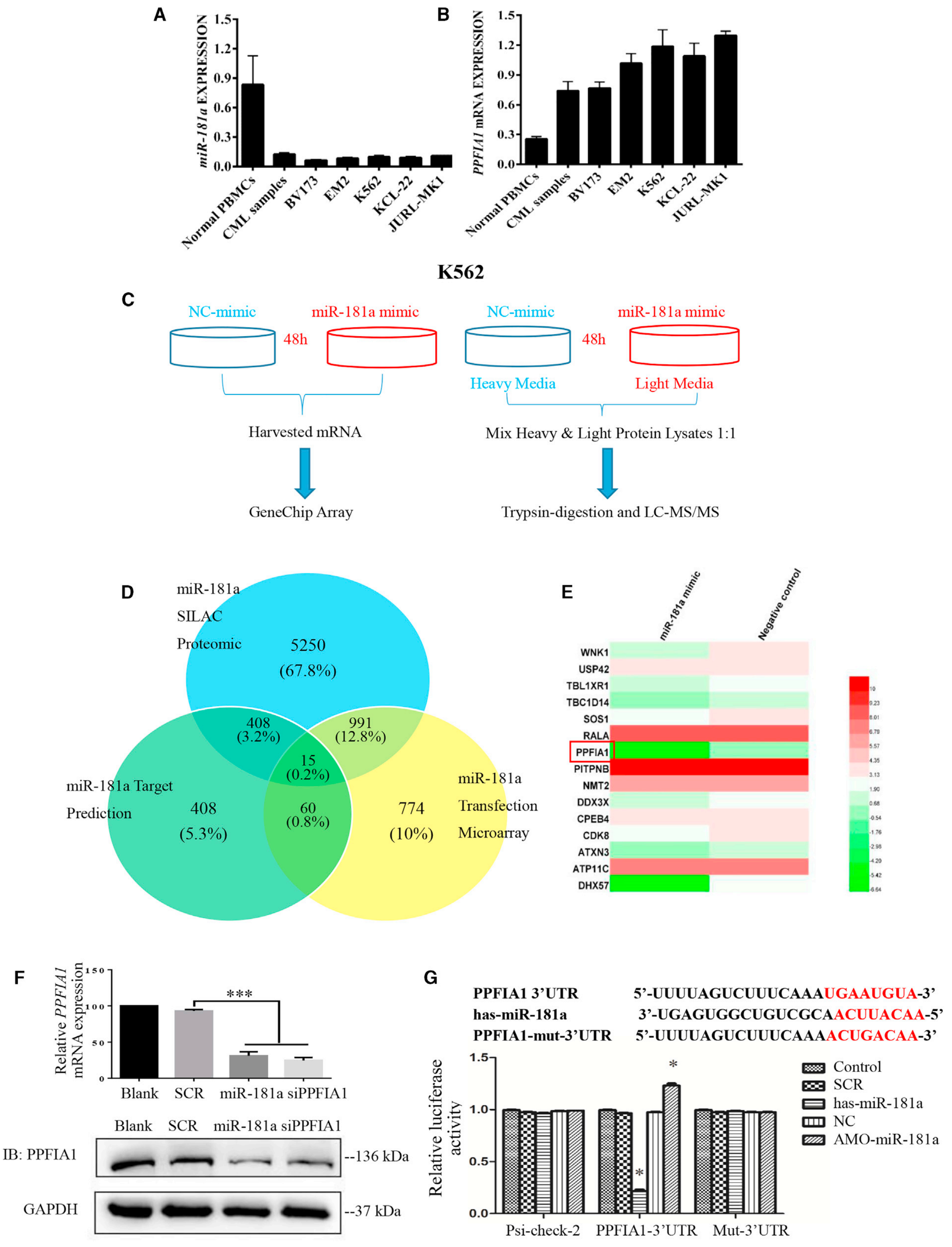

(legend on next page)

targets of miR-181a, and thereby determine the mechanisms of tumor suppression and downstream molecules by miR-181a, we carried out stable isotrope labeling by/with amino acids in cell culture (SILAC)-based proteomic profiling, along with miRNA prediction and GeneChip analysis, through overexpression of miR-181a mimic in K562 cells. This multi-omics approach provided new insights and could be used as a general strategy to study the targets of individual miRNAs. Further investigation of a subset of downregulated candidate targets confirmed them as novel direct targets of miR-181a. Among the candidate targets, protein tyrosine phosphatase, receptor type, f polypeptide (PTPRF), leukocyte common antigen (LAR) interacting protein (liprin), alpha 1 (PPFIA1) may be an important oncogene in CML.

PPFIA1 is a member of the liprin family, and its encoding gene maps to the 11q13 amplification region,[15] which is one of the most common amplicons in multiple epithelial cancers, including breast cancers,[16] head and neck squamous cell carcinomas (HNSCCs),[17] and oral squamous cell carcinomas (OSCC).[18] Depletion of PPFIA1 results in increased invasion of HNSCC cells.[17] PPFIA1 is frequently co-amplified along with cyclin D1 in oral carcinomas and could be a useful biomarker, as well as a novel target for specific gene therapy.[18] PPFIA1 is required for the functions of ING4 to suppress cell spreading and cell migration in colon carcinoma cell.[19] However, its role and molecular basis for PPFIA1 in CML has not previously been established.

The overarching goal of the present study was to uncover the anti-CML role of miR-181a and illustrate the effects of miR-181a's candidate target. PPFIA1, a direct target of miR-181a identified by multi-omics, has played a central position within a possible regulation miR-181a/PPFIA1/PARP1/nuclear factor κappa B (NF-κB)-P65/KIT axis controlling the expression of KIT. Interestingly, many of the pharmacologic agents that were used to target KIT expression are already applied in the clinic. Our investigation has revealed that targeting PPFIA1 and PARP1 in the miR-181a/PPFIA1/PARP1/NF-κB-P65/KIT axis could attain significant and durable anti-leukemic activity in CML.

RESULTS

PPFIA1, a Direct Target Gene of miR-181a in CML

To determine miR-181a expression levels in healthy blood cells, human CML samples, and CML cell lines, we extracted total RNA and then performed qPCR assays. As shown in Figure 1A, miR-181a expression was downregulated in human CML samples and CML cell lines, compared with its expression in healthy human peripheral blood mononuclear cells (PBMCs), indicating that downregulation of miR-181a may have an important role in leukemogenesis. To identify miR-181a targets that related to its effects of anti-CML, we combine SILAC-liquid chromatography-tandem mass spectrometry (LC-MS/MS) and GeneChip analysis to systematically investigate the impact of overexpression miR-181a in CML cell line K562 (Figure 1C). Using SILAC-LC-MS/MS analysis, we identified that over 6,000 proteins were downregulated after reintroduction of miR-181a in K562. Next, GeneChip analysis was performed after transfection with 100 nM miR-181a mimic or negative control (NC) for 48 h in K562 cells, and we identified more than 1,500 genes' downregulation in the miR-181a mimic transfection group. To further screen the potential targets of miR-181a in K562 cells, we identified 15 candidate miR-181a target genes by combining miRNA prediction, SILAC-LC-MS/MS, and GeneChip methods (Data S1, S2, and S3). *PPFIA1*, 1 of 15 candidates, was selected for further analysis (Figures 1D and 1E). Next, we tested whether miR-181a can downregulate *PPFIA1* mRNA in K562 cells. Real-time RT-PCR demonstrated that *PPFIA1* mRNA levels in K562 cells transfected with either miR-181a mimic or PPFIA1 small interfering RNA (siRNA) decreased substantially, compared with the negative control group. Subsequently, we evaluated the effect of miR-181a on the expression of PPFIA1 protein by western blotting. Overexpression of miR-181a and PPFIA1-siRNA resulted in reduced PPFIA1 protein levels (Figure 1F). To determine whether the 3′ UTR of *PPFIA1* contained functional miR-181a target sequence(s), and whether direct interaction occurred between these and miR-181a, we constructed a pair of dual-luciferase reporter plasmids containing two tandem putative miR-181a recognition sequences from the 3′ UTR of the *PPFIA1* mRNA and the mutated versions of these sequences immediately downstream of the luciferase gene (psi-CHECK-PPFIA1-3′ UTR and psi-CHECK-PPFIA1-mut-3′ UTR, respectively). Using these plasmids, we performed dual-luciferase reporter assays. As shown in Figure 1G, transfection with miR-181a mimic significantly decreased the luciferase activity from psi-CHECK-PPFIA1-3′ UTR, but not that from psi-CHECK-PPFIA1-mut-3′ UTR, in 293T cells. Together, these data suggest that PPFIA1 may be a direct target of miR-181a in K562 cells.

The Effects of PPFIA1 or miR-181a on the Malignant Progression of CML

Next, we compared PPFIA1 mRNA level in CML cells with PBMCs. As shown in Figure 1B, PPFIA1 level was higher in the tested CML cells and human CML samples. We also determined whether reducing *PPFIA1* mRNA levels by treatment with either PPFIA1

Figure 1. *PPFIA1*, a Direct Target Gene of miR-181a in CML

(A) miR-181a was downregulated in CML cells. The qPCR analysis of miR-181a in normal human PBMCs, human CML samples, and CML cell lines. (B) PPFIA1 mRNA was upregulated in CML cells. The qPCR analysis of PPFIA1 mRNA in normal human PBMCs, human CML samples, and CML cells. (C) Experimental design for microarray analysis or SILAC-LC-MS/MS analysis. (D) 15 candidate target genes of miR-181a were identified by combining miRNA predictions, miR-181a transfection microarray, and SILAC-LC-MS/MS analysis. (E) Heatmap showed the different expression of 15 candidate target genes in miR-181a transfection microarray. (F) PPFIA1-siRNA and miR-181a mimics (100 nM) inhibited the mRNA and protein expression levels of PPFIA1. (G) Human PPFIA1-3′ UTR and its miR-181a target site predicted by TargetScan software. miR-181a-directed repression of Renilla luciferase reporter genes containing PPFIA1-3′ UTR segments. The data were presented as the mean ± SD obtained from at least three independent experiments. Significance was determined by Student's t test, *$p < 0.05$ versus NC control. Mut, contains 7-base mutation at the miR-181a target seed region.

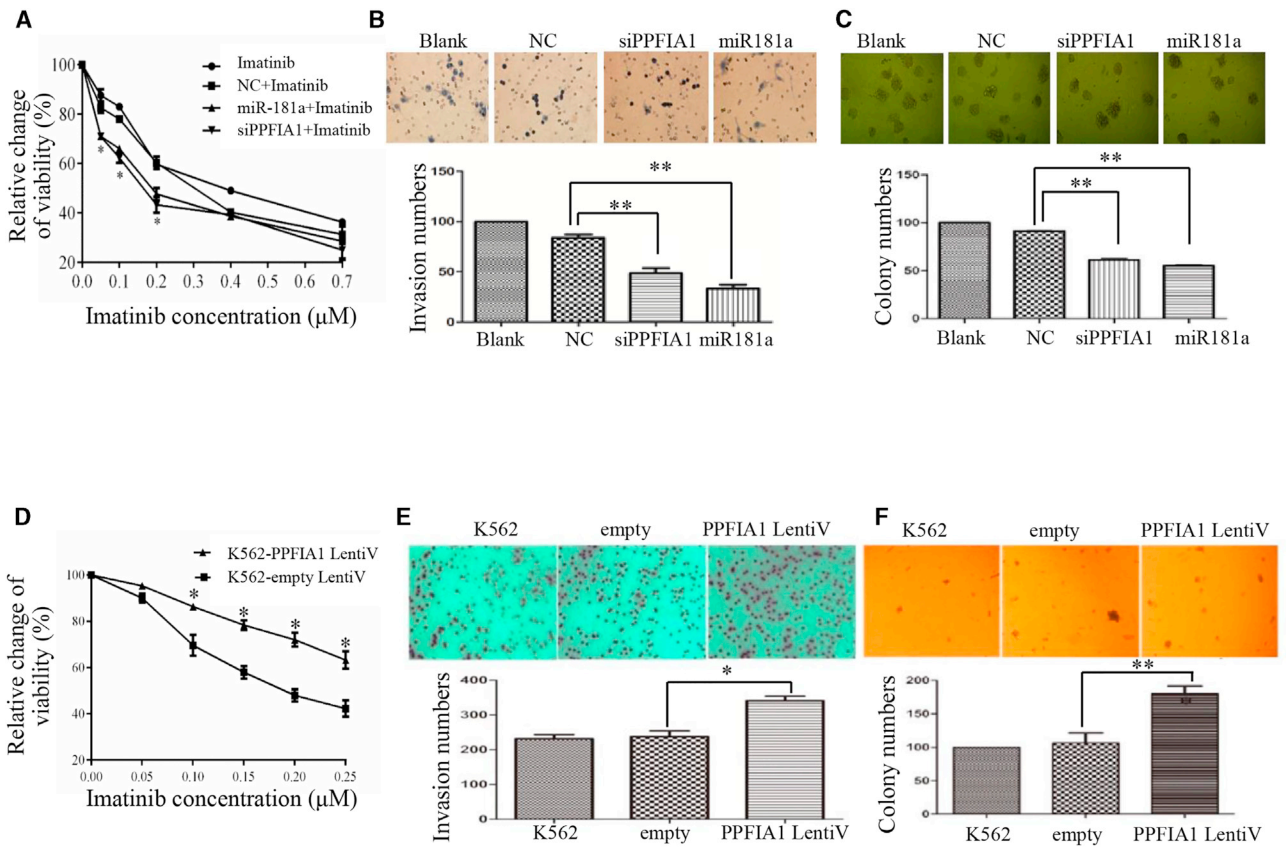

Figure 2. The Effects of PPFIA1 or miR-181a on the Malignant Progression of CML

(A) Downregulation of PPFIA1 by transfecting with PPFIA1-siRNA and miR-181a mimics increased the sensitivities of STI571 in K562 cells. (B) Downregulation of PPFIA1 inhibited the invasion ability in K562 cells. (C) Downregulation of PPFIA1 by targeting PPFIA1-siRNA or miR-181a mimics inhibited the colony-formation ability in K562 cells. (D) Overexpression of PPFIA1 decreased the sensitivities of STI571 in K562 cells. (E) Overexpression of PPFIA1 promotes the invasion ability in K562 cells. (F) Overexpression of PPFIA1 promotes the colony-formation ability in K562 cells. The data were presented as the mean ± SD obtained from at least three independent experiments. Significance was determined by Student's t test, *p < 0.05 versus NC control or empty vector.

siRNA or miR-181a mimic could attenuate the CML malignant behavior. Both miR-181a mimic and PPFIA1 siRNA transfection groups effectively increased K562 cell sensitivity to IM treatment in a concentration-dependent manner (Figure 2A). Transwell assays also showed that the invasion ability of CML cells was significantly reduced by treatment with either miR-181a mimic or PPFIA1 siRNA (Figure 2B). Finally, the colony-forming ability of K562 cells transfected with miR-181a mimic or PPFIA1 siRNA was significantly reduced compared with cells transfected with the NC group ($p < 0.05$) (Figure 2C).

To test whether the high expression of PPFIA1 in CML is associated with therapeutic response, we analyzed by 3-(4,5-dimethylthiazol-2-yl)-2,5-diphenyl-tetrazolium bromide (MTT) assay the IM sensitivity of K562 cells overexpressing PPFIA1 after treatment of cells with IM for 48 h. The results showed that overexpression of PPFIA1 significantly inhibited the cell-killing effect of IM on K562 cells, as indicated by increased cell viability (Figure 2D). Cell invasion is a key malignant behavior associated with tumorigenesis and cancer metastasis. To detect whether increased PPFIA1 levels could contribute to the malignant phenotypes of K562 cells, we conducted Transwell cell invasion assays using BioCoat Matrigel. Overexpression of PPFIA1 significantly enhanced cell invasion ($p < 0.05$) (Figure 2E). Next, we investigated the effect of PPFIA1 on K562 cell clonogenicity using colony-forming assays to determine the neoplastic capacity of cells. As shown in Figure 2F, overexpression of PPFIA1 significantly increased the number of K562 cell colonies, compared with controls ($p < 0.05$). Together, these results indicate that PPFIA1 and miR-181a expression levels are correlated with malignant phenotypes in K562 cells.

The Inhibitory Effects of PPFIA1 siRNA on CML Xenograft Murine Model

To investigate the therapeutic potential of targeting PPFIA1 with PPFIA1-siRNA, we intravenously injected NOD-*Prkdc*^{scid}*IL2rg*^{tm1}/Bcgen (NSG) mice with 10^6 luciferase-labeled K562 cells, and the effects of siRNA treatment were monitored by bio-imaging. Five days after injection with K562-luciferase cells, NSG mice were treated with 10 nmol PPFIA1-siRNA or NC siRNA control for seven times

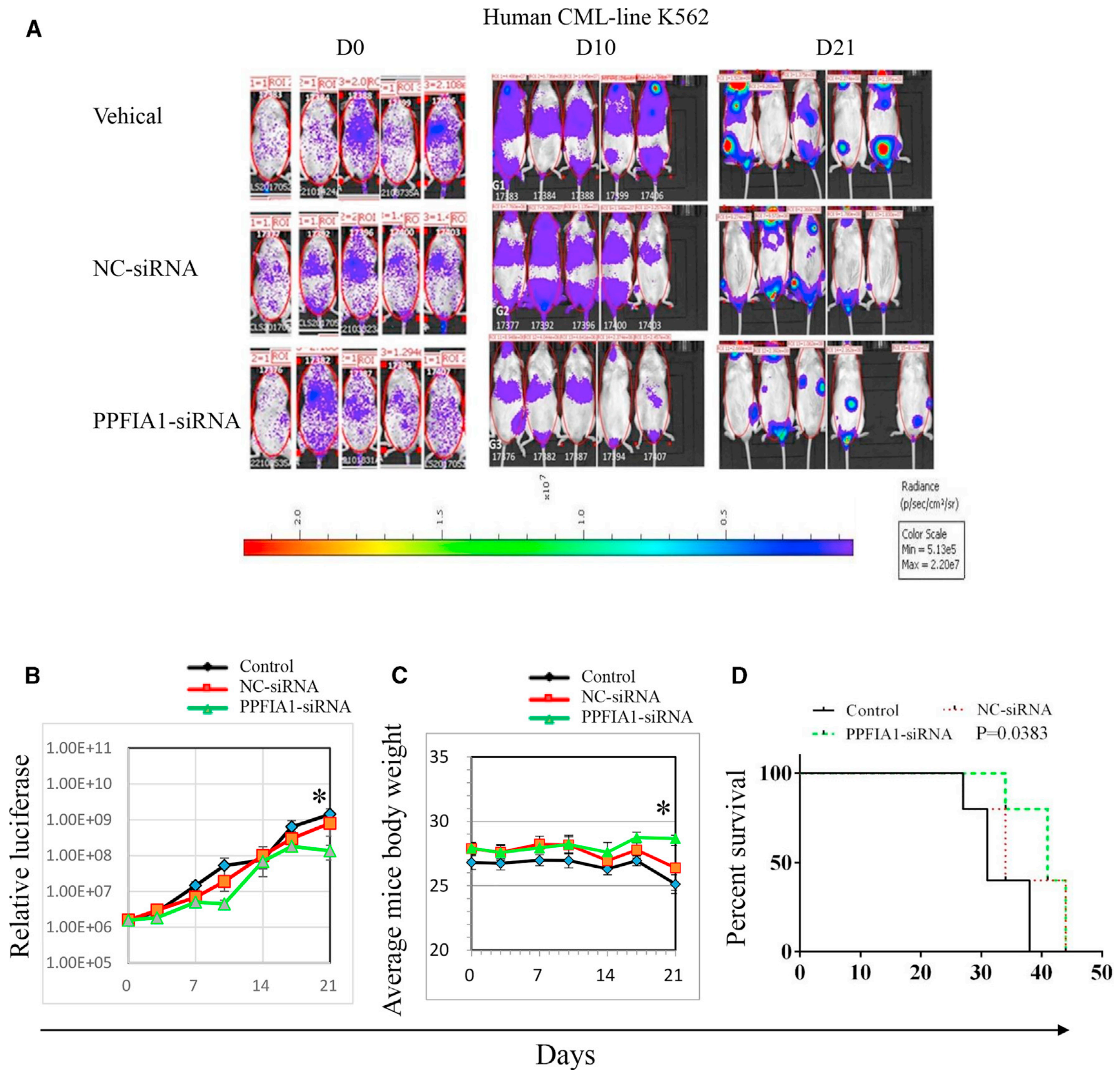

Figure 3. The Inhibitory Effects of PPFIA1 siRNA on CML Xenograft Murine Model

(A) NSG nude mice were transplanted with K562-luciferase cells and after 5 days treated with saline control, 10 nmol NC-siRNA, or 10 nmol PPFIA1-siRNA. Images showing the tumor burden of K562-luciferase cells in NSG mice after treatment (n = 5/group). (B) Luciferase changes of NSG mice treated with saline control, NC-siRNA, or PPFIA1-siRNA. Significance was determined by Student's t test: PPFIA1-siRNA to saline control (p = 0.046), PPFIA1-siRNA to NC-siRNA (p = 0.049). (C) Body weight changes of NSG mice treated with saline control, NC-siRNA, or PPFIA1-siRNA. Significance was determined by Student's test: PPFIA1-siRNA to saline control (p = 0.001), PPFIA1-siRNA to NC-siRNA (p = 0.02). (D) Survival analysis of NSG mice with CML treated with saline control, NC-siRNA, or PPFIA1-siRNA. Significance was determined by Student's t test, *p < 0.05 versus NC control. Survival curves were compared by log rank test (p = 0.0383).

every other day. PPFIA1 siRNA significantly inhibited the growth of K562-luciferase cells in mice compared with those treated with NC-siRNA and vehicle controls on day 21 (Figure 3A); the relative luciferase levels are presented in Figure 3B. The relative luciferase level in mice receiving NC-siRNA treatment at the time of tumor inoculation was 1.58e+6; this then increased to 8.01e+8 on day 21, whereas it increased from 1.56e+6 to 1.35e+8 in the mice receiving PPFIA1-siRNA treatment (p = 0.049). These results indicate that targeting PPFIA1 using PPFIA1-siRNA can inhibit the growth of K562-luciferase cells *in vivo*. Body weight is considered to be an indicator of cancer cachexia in animal models. The average body weight of mice receiving NC-siRNA treatment at the time of tumor inoculation was 27.9 g, and this reduced to 26.4 g on day 21, whereas it increased from 27.9 to 28.4 g in mice receiving PPFIA1-siRNA treatment (Figure 3C) (p = 0.02). Cytospins of histopathology of spleen and liver sections from mice receiving K562-luciferase cells exhibited lower PPFIA1 and PARP1 protein expression than vehicle control engrafted mice (Figures S1 and S2). NSG mice treated with PPFIA1 siRNA survived for longer periods than those administered with NC-siRNA and blank control groups (Figure 3D) (p = 0.0383). These results indicate

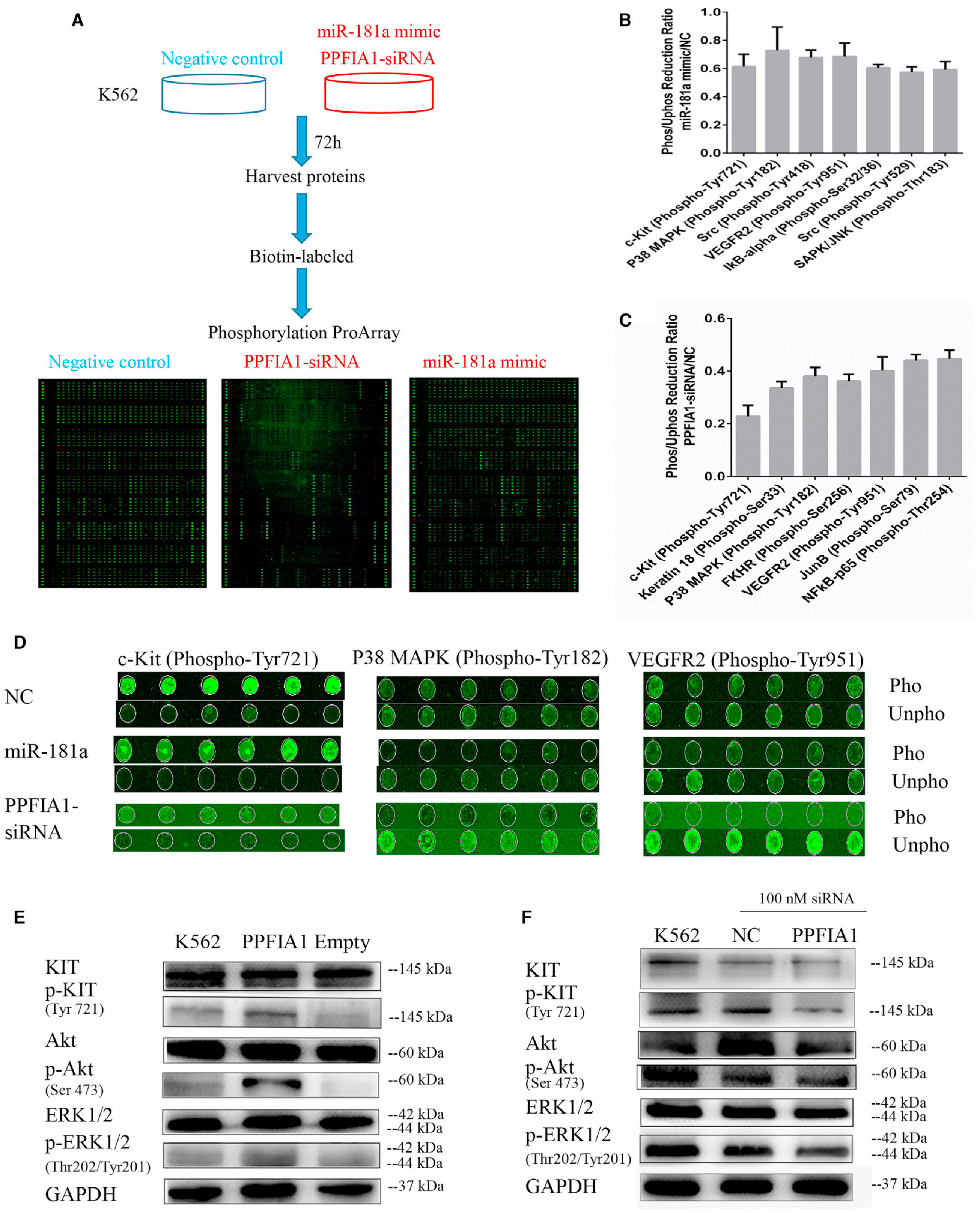

(legend on next page)

that PPFIA1 siRNA may be a promising drug for the treatment of CML.

PPFIA1-siRNA Reduces KIT Phosphorylation Level as Detected by Phospho-Array Assay

To understand the mechanisms underlying the role of PPFIA1 in enhancing invasion, and clonogenicity of CML cells, we performed phospho-array assays. K562 cells transfected with 100 nM NC, miR-181a mimic, and PPFIA1 siRNA for 72 h were subjected to analysis of their protein phosphorylation profiles. Phosphorylation ratios were calculated to determine the differences between two samples (PPFIA1-siRNA/NC and miR-181a mimic/NC) at 248 site-specific phosphorylation sites implicated in cancer signaling (Figure 4A; Data S4). In the miR-181a mimic transfection group, phosphorylation levels were reduced at 23 protein sites, and 7 of them were reduced by $\geq 30\%$ normalized to the NC transfection group (Figure 4B). PPFIA1 siRNA transfection reduced the phosphorylation of 60 protein sites, and 8 of them were decreased by >50% (Figure 4C). Notably, a comparison of the phosphorylation-reduced ratio allowed us to discern three phosphorylation targets whose phosphorylation was reduced commonly by miR-181a mimic and PPFIA1-siRNA treatments, including KIT Tyr721, P38 mitogen-activated protein kinase (MAPK) Tyr182, and vascular endothelial growth factor receptor 2 (VEGFR2) Tyr951 sites (Figure 4D).

Notably, KIT has been implicated in CML pathogenesis, and KIT-expressing BCR-ABL-transduced murine myeloid cells are less sensitive to tyrosine kinase inhibitors.[4,5] Therefore, we postulated that PPFIA1, the target of miR-181a, may promote malignant phenotypes through activating the KIT signaling pathway in K562 cells. Phosphorylated KIT Tyr721 expression was markedly increased in K562 cell lines stably overexpressing PPFIA1 (Figure 4E), whereas using PPFIA1-siRNA to knock down its expression resulted in the opposite effect (Figure 4F). The same results can also be observed in Kcl-22 cells (Figure S3). Recent studies have shown that KIT can promote the proliferation and invasion of cancer cells through activating Akt and extracellular regulated protein kinase 1 (ERK1)/2 pathways. As expected, similar results were attained for the expression of phosphorylated ERK1/2 and Akt. Collectively, these data suggest that PPFIA1 may have an oncogenic role through activation of the KIT pathway in CML.

PARP1, an Important Downstream Molecule of PPFIA1 and BCR/ABL

To explore the relationship between PPFIA1 and KIT, we ectopically overexpressed FLAG-PPFIA1 in K562 cells and performed BCR/FLAG immunoprecipitation (IP) assays. The IP products of BCR and FLAG affinity purification were separated by SDS-PAGE and analyzed by mass spectrometry (Data S5 and S6). PARP1 was associated with both exogenous PPFIA1 and endogenous BCR in K562 cells (Figure 5A). To test for direct binding of PPFIA1-PARP1 and ABL-PARP1, we performed computer-based molecular docking and surface plasmon resonance imaging (SPRi). The molecular docking analyses indicated that the PARP1 catalytic domain can bind to PPFIA1 (PDB: 3TAC) and ABL1 (PDB: 5HU9) (Figures 5B and 5C). The domain structures of ABL1 were obtained from PDB (F-actin PDB: 1zzp; SH2 PDB: 4J9I), and the results of molecular docking analysis showed the F-actin domain of ABL1 can dock with the PARP1 catalytic domain (Figure 5D), whereas the SH2 domain cannot (Figure S4). Because the BCR/ABL fusion protein was unattainable, we purified each domain of ABL and performed an SPRi assay to determine the relationship between PARP1 and BCR/ABL. The results of SPRi confirmed that the F-actin domain of ABL1 can bind to PARP1, with a K_d (M) of $1.36e{-}8$, which is consistent with the results of molecular docking analysis (Figure 5E).

Interestingly, in addition to the molecular docking analysis demonstrating that PARP1 can interact with PPFIA1, IP experiments were also able to identify this interaction. As shown in Figures 5F–5I, PARP1 interacted with PPFIA1 and ABL1, and may serve as an important downstream molecule of PPFIA1 or BCR/ABL. Based on these results, we hypothesized that PARP1 mediates the relationship between PPFIA1 and KIT overexpression.

PARP1 Promotes KIT Expression by Activating NF-κB-P65 Transcription

PARP1 forms branched poly(ADP) ribose (PAR) polymers on several proteins involved in DNA repair and transcription, including PARP1 as a key component of the NF-κB transcription regulatory complex.[20] Therefore, we hypothesized that PPFIA1 contributes to leukemia growth through an axis involving PARP1, P65, and KIT. Consistent with our hypothesis, forced *PARP1* expression in K562 cells led to upregulation of P65 expression levels, whereas decrease of PARP1 expression using olaparib (a PARP1 inhibitor [PARPi]) led to downregulation of P65 (Figures 6A–6C). Furthermore, forced P65 expression in K562 cells led to KIT mRNA and protein upregulation, whereas decreased P65 expression using P65-siRNA led to downregulation of KIT levels (Figures 6D–6F). Together, these data suggest that PPFIA1 participates in modulation of KIT levels by interacting with PARP1, and PARP1 regulates the transcriptional activity of

Figure 4. PPFIA1-siRNA Reduces KIT Phosphorylation Level as Detected by Phospho-Array Assay

(A) Experimental design to detect the phosphorylative signal proteins in K562 cells transfected with PPFIA1-siRNA or miR-181a mimics. (B and C) Phosphorylative signal proteins in K562 cells transfected with PPFIA1-siRNA (B) and miR-181a mimic (C) were comprehensively investigated using an antibody microarray system compared with control. The phosphorylation ratio (Phos Ratio) was used as the modulation difference of phosphorylation sites between two samples and was computed as follows: phosphorylation ratio = phospho value/non-phosphorylated value. (D) The phosphor-fluorescent and unphospho-fluorescent images of KIT Tyr721, P38 MAPK Tyr182, and VEGFR2 Tyr951 sites. (E) Overexpression of PPFIA1 increased the expression of phosphorylated KIT, Akt, and ERK1/2 proteins in K562 cells. (F) Transfection of PPFIA1-siRNA for 48 h attenuated the expression of phosphorylated KIT, Akt, and ERK1/2 proteins in K562 cells. The data were presented as the mean ± SD obtained from at least three independent experiments.

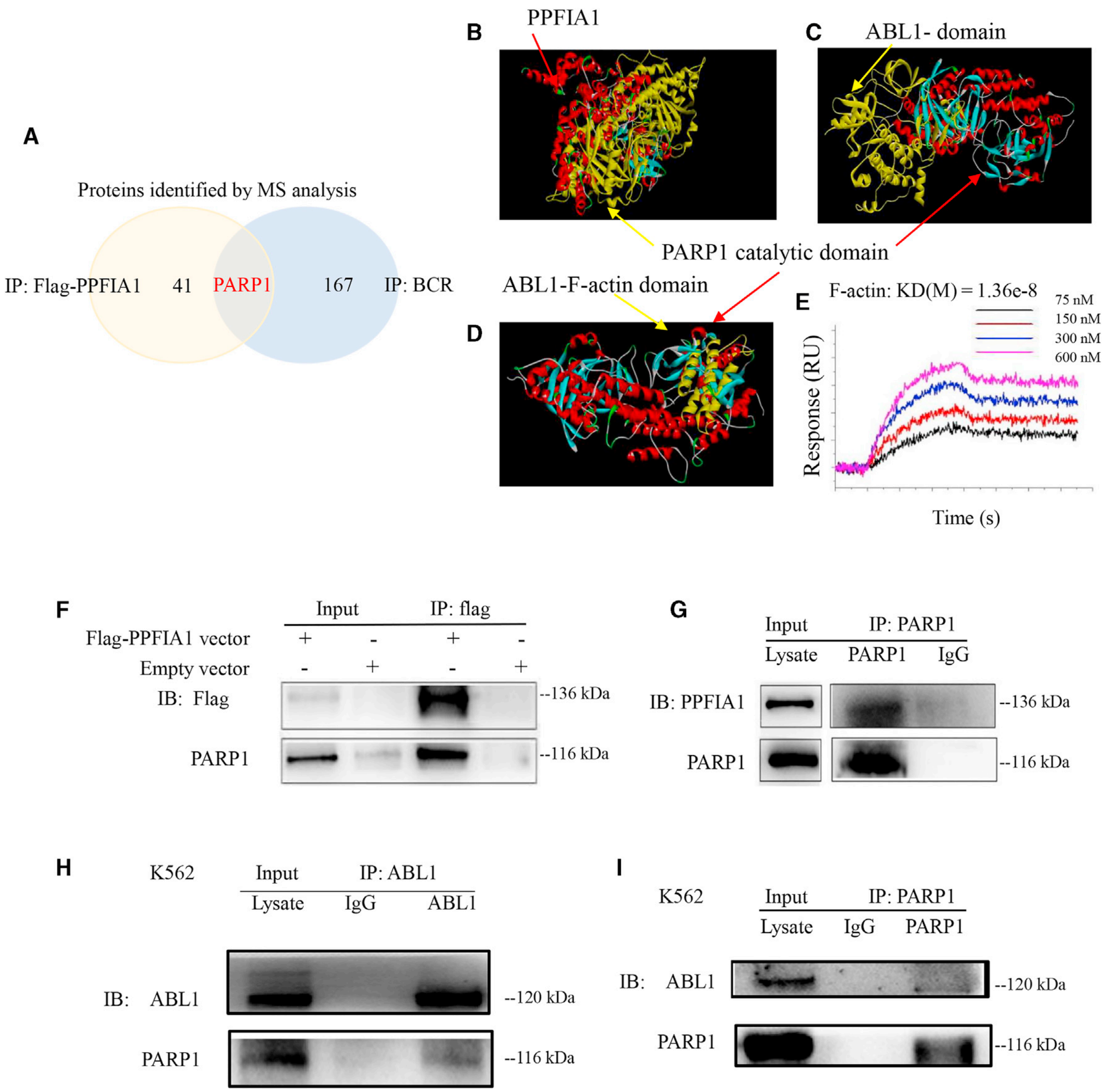

Figure 5. PARP1, an Important Downstream Molecule of PPFIA1 and BCR/ABL

(A) K562 or K562-Flag PPFIA1 cells were used to perform BCR/FLAG IP assays. The IP products from BCR and FLAG affinity purification were separated by SDS-PAGE and analyzed by mass spectrometry. The PARP1 was both related to exogenous PPFIA1 and endogenous BCR in K562 cells. (B) The molecular docking of PPFIA1 with PARP1 by using Discovery Studio 4.5. The molecular docking of ABL1-PARP1 (C) or F-actin-PARP1 (D) by using Discovery Studio 4.5. (E) The binding analysis of F-actin-PARP1 was performed in surface plasmon resonance imaging (SPRi) binding assay. (F) FLAG-immunoprecipitates were immunoblotted with anti-Flag and anti-PARP1. PARP1 was immunoprecipitated with FLAG affinity purification. (G) PARP1-immunoprecipitates were immunoblotted with anti-PPFIA1 and anti-PARP1. PPFIA1 was immunoprecipitated with anti-PARP1. (H) ABL1-immunoprecipitates were immunoblotted with anti-ABL1 and anti-PARP1. PARP1 was immunoprecipitated with anti-ABL1. (I) PARP1-immunoprecipitates were immunoblotted with anti-ABL1 and anti-PARP1. ABL1 was immunoprecipitated with anti-PARP1.

NF-κB-P65. PPFIA1/PARP1/NF-κB-P65/KIT appears to constitute a regulatory axis in CML.

Next, to explore the therapeutic potential of targeting PARP1 in CML, we investigated the effect of olaparib on K562 cells stably expressing PPFIA1. Olaparib notably suppressed the colony-forming ability of cells transformed with PPFIA1-expressing lentivirus (Figure 6G). Having demonstrated the biological relevance of the PPFIA1/PARP/P65/KIT axis *in vitro*, we next tested whether PPFIA1 has an oncogenic effect on Baf3-P210-driven leukemogenesis *in vivo*. We generated a Baf3-P210 construct, which stably expresses PPFIA1 and allows cell selection with puromycin. Given that PARPis can slow AML cell growth and affect the differentiation of AML1-ETO- and PML-RARA-transformed mouse cells, we postulated that olaparib may repress CML progression *in vivo*. To test this hypothesis, Baf3-P210 cells (5×10^6/mouse) were engrafted into BALB/c mice

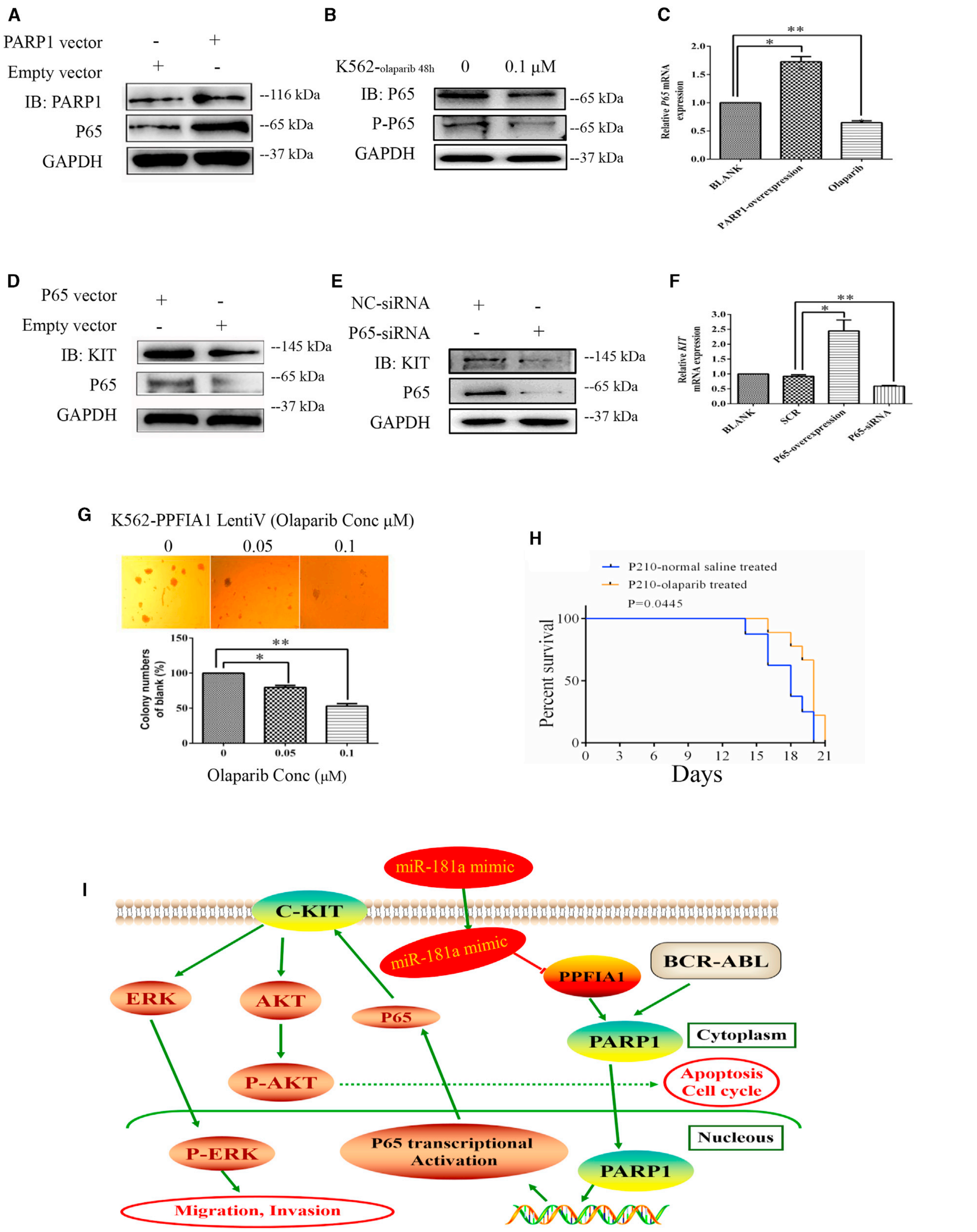

(legend on next page)

irradiated with 3 Gy X-ray. Engrafted mice were treated with olaparib or stroke-physiological saline solution alone (vehicle control) daily for 7 days. Animals treated with olaparib survived for longer periods than those receiving vehicle control (Figure 6H) (p = 0.0445). A summary diagram outlining the regulatory network discussed above is presented in Figure 6I. These results indicate that PARPis represent a promising therapeutic approach to overcome CML.

DISCUSSION

Emerging evidence demonstrates that altered miRNA expression, including that of miR-181, is involved in drug resistance in CML patients.[21] Meta-analyses have indicated that low miR-181a expression is significantly associated with poor survival outcomes in hematological malignancies,[9] and our results indicate that mir-181a expression is much lower in CML cell lines than normal controls. A key mechanism for miRNA-mediated regulation of targets is repression of protein synthesis, which can occur with or without alteration of mRNA transcript abundance. To supplement bioinformatics and GeneChip analyses, in this study we performed SILAC-based quantitative proteomics. The use of this multi-omics approach to narrow the potential target genes indicated that PPFIA1 may be a direct target of miR-181a in K562 cells. This type of multi-omics approach has the potential to advance biological research in other contexts.

The overexpression of PPFIA1 in different tumor types is generally associated with a prognosis of more aggressive cancer development.[22–24] Further research is required to determine the roles of polypeptide-interacting protein (PTP)-interacting proteins, particularly PPFIA1, in CML. Here we found that PPFIA1 plays an important role in CML tumorigenesis and hypothesize that targeting PPFIA1 expression may show anti-CML activity and confer a therapeutic benefit in CML patients. We tested this hypothesis by synthesizing a PPFIA1-siRNA formulated in 5′-cholesterol. The mechanism of improved distribution and cellular uptake of siRNA through cholesterol conjugation was demonstrated in a recent study;[25] cholesterol-siRNA conjugates appear to be incorporated into circulating lipoprotein particles and are efficiently internalized by hepatocytes via a receptor-mediated processes.[26,27] Treatment with PPFIA1-siRNA significantly decreased the luciferase signal in NSG mice xenografted with K562-luciferase cells and exhibited strong antileukemic activity, leading to improved survival. RNAi is an emerging therapeutic strategy that has been widely investigated. Despite a few promising clinical trials, effective delivery of siRNA *in vivo* remains a pivotal challenge in translating RNAi into the clinic as a conventional treatment option. The approach used in this study both enabled us to gain biological insight into the role of PPFIA1 in CML and laid the foundation for a future siRNA-based therapeutic approach targeting PPFIA1 in a subpopulation of leukemia cells. Our study reveals that PPFIA1 could be involved in the pathogenesis of CML and may be a potential target for the treatment of this malignancy.

It is established that BCR/ABL can activate multiple pathways involved in the regulation of cell proliferation and apoptosis, including phosphatidylinositol 3-kinase (PI3K)/Akt,[28] Ras,[29,30] STAT5,[31] and Raf/MEK/ERK.[32] PPFIA1 may activate several signaling pathways to support its oncogenic role, similar to BCR/ABL. We used a phospho-antibody microarray-based proteomics approach and identified multiple pro-metastatic proteins (including KIT Tyr721). Binding of SCF to KIT activates multiple signal transduction pathways, including PI3K/Akt, p44/42 MAPK, and STAT.[33] Akira Yasuda et al.[34] elucidated the role of PI3K/Akt and p44/42 MAPK in SCF-enhanced proliferation and invasion of colorectal cancer cells. Certain human cancers, including CML, are characterized by aberrant KIT tyrosine kinase activity, and KIT is implicated in CML pathogenesis. KIT signaling can substitute for BCR/ABL tyrosine kinase activity to activate survival signals, and governs the differential sensitivity of mature or primitive CML progenitors to tyrosine kinase inhibitors.[4,5] In this study, we showed that the PPFIA1 protein can activate several signaling pathways, including those involving KIT, Akt, and ERK1/2 in CML cells. Because KIT expression must be inhibited to enable apoptosis induced by BCR/ABL inhibitors in CML cells, our findings provide a rationale for therapeutic targeting of PPFIA1 levels to overcome aberrant phospho-KIT activity and induce significant anti-CML effects.

The current study was designed to discover mechanisms that regulate KIT expression, so that treatment strategies can be developed to circumvent the drawbacks of therapy with tyrosine kinase inhibitors (TKIs). Our investigation indeed led to the identification of a regulation axis that deregulates KIT expression, impacts leukemogenesis, and is pharmacologically targetable. We suggest that PARP1 may be an important molecule downstream of BCR/ABL and PPFIA1. PARP1 plays an important role in modulating the cellular responses to DNA damage and transcription.[35] The expression of PARP1 is upregulated in BCR/ABL^+ cells as part of a non-homologous end joining DNA repair pathway (ALT NHEJ) and can serve as biomarker to

Figure 6. PARP1 Promotes KIT Expression by Activating NF-κB-P65 Transcription

Forced expression of PARP1 enhanced P65 protein expression level (A) and downregulated PARP1 expression by olaparib-inhibited P65 protein expression level in K562 cells (B). (C) Forced expression of PARP1 enhanced *P65* mRNA expression level and downregulated PARP1 expression by olaparib-inhibited *P65* mRNA expression level in K562 cells. Forced expression of P65 enhanced KIT protein expression level (D), and downregulated P65 expression by P65-siRNA transfection inhibited KIT protein expression level in K562 cells (E). (F) Forced expression of P65 enhanced *KIT* mRNA expression level, and downregulated P65 expression by P65-siRNA transfection inhibited *KIT* mRNA expression level in K562 cells. (G) Olaparib inhibited the colony-formation abilities of K562 cells with stable overexpression of PPFIA1. (H) Olaparib administered at the dose of 50 mg/kg daily increased survival duration in mice engrafted with Baf3-P210 cells compared with untreated Baf3-P210 cells engrafted controls (n = 7/group). Curves were compared by log rank test (p = 0.0445). (I) Summary diagram describes the function of PPFIA1/PARP1/KIT/NF-κB-P65 network in CML cell lines. The data were presented as the mean ± SD obtained from at least three independent experiments. Significance was determined by Student's t test, **p < 0.01, *p < 0.05 versus NC control.

identify a subgroup of CML patients who may be candidates for therapies that target the ALT NHEJ pathway when treatment with TKIs has failed.[36] Recent evidence also supports a role for PARP1 as a transcriptional co-regulator, with the enzyme implicated in the control of NF-κB expression.[37] We found that PPFIA1 directly interacted with PARP1, suggesting that PARP1 has an important regulatory role in PPFIA1-related oncogenic and signaling pathway functions. We showed that gain-of-function PARP1 expression in K562 led to constitutive P65 upregulation, which is likely to be an initial step in increased KIT expression. Activated PARP1 is required to trigger I-kappa kinase (IKK)γ SUMOylation, which in turn permits IKK and NF-κB activation, as well as NF-κB-regulated resistance to apoptosis.[38] Others have demonstrated that upregulation of NF-κB can enhance target transactivation, such as KIT expression.[39] Esposito et al.[20] described the potential utility of PARPi-induced synthetic lethality for leukemia treatment and revealed a novel molecular mechanism governing PARPi sensitivity in AML. We investigated the effect of olaparib, a PARPi, in BALB/c mice xenografted with Baf3-P210 cells. Olaparib showed strong anti-leukemia activity and led to improved survival of mice. Therefore, we hypothesize that PPFIA1 or PARP1 might be central within a regulatory axis controlling KIT expression.

In conclusion, our investigation has identified a targetable miR-181a/PPFIA1/PARP1/NF-κB-P65/KIT regulatory axis that modulates KIT expression in CML. A multi-omics approach showed that miR-181a downregulation results in aberrant activation of PPFIA1, which interacts with PARP1, an important downstream molecule of PPFIA1 and BCR/ABL, to further trans-activate KIT expression through activation of NF-κB-P65. Therapeutic targeting of PPFIA1 and PARP1 in the miR-181a/PPFIA1/PARP1/NF-κB-P65/KIT axis can control the expression of KIT in CML. An attractive aspect of our study is the potential for the translation of our findings into clinical trials by targeting multiple molecular in the regulatory axis, and PPFIA1/PARP1 appears to have an oncogenic role after downregulation of miR-181a in CML.

MATERIALS AND METHODS

Materials

Antibodies; cell lines; mouse models; chemicals, recombinant proteins, and plasmids; and oligonucleotides used in this study are listed in Tables S1–S5, respectively.

Primary Cells

Healthy peripheral blood or CML BM samples were obtained from healthy adult donors in Guangdong Provincial Emergency Hospital/the Guangdong Second Provincial General Hospital after written informed consent according to institutional guidelines and Declaration of Helsinki principles.

Cells Culture and Transfection

Cells were grown in suspension in RPMI 1640 medium supplemented with 10% fetal bovine serum (FBS). All culture media were supplemented with 100 U/mL penicillin and 100 μg/mL streptomycin. Lentiviral infection to establish K562 cell lines stably expressing PPFIA1, 3-FLAG-PPFIA1, P65, and PARP1 was performed according to the manufacturer's instructions (FulenGen, Guangzhou, China). Normal peripheral blood samples were obtained from three healthy donors. Prior informed consent was obtained from the donors with the approval of the institutional review board. All RNA mimic and siRNA (50, 75, 100, or 125 nM) were transfected into CML cells using Lipofectamine 2000, according to the manufacturer's instructions.

Real-Time PCR Assay

Total RNA from CML cells lines and primary samples was extracted using TRIzol (Invitrogen). After reverse transcription, the mRNAs were detected using SYBR-Green real-time PCR assays. mRNA expression levels were normalized to those of *GAPDH*, and the fold-change of mRNA levels was calculated using the $2^{-\Delta\Delta CT}$ method.

PPFIA1 3′ UTR Plasmids and Reporter Assays

Two oligonucleotides, each containing a *PPFIA1* miR-181a target site, were synthesized as 5′-CCG ctcgag AGTCTCCT GTTGTTTACC CACAC-3′ (sense strand) and 5′-ATAAGAAT gcggccgc TCATCT CCTGGCTGC TTTATTC-3′ (antisense strand), and amplified by PCR. The resulting PCR product was cloned into the psiCHECK-2 vector (Promega, Madison, WI, USA) using XhoI and NotI sites (lowercase letters) immediately downstream of the Renilla luciferase reporter gene to generate psi-CHECK-PPFIA1-3′ UTR. To clone psi-CHECK-PPFIA1-mut-3′ UTR containing mutant recognition sequences, two oligonucleotides, 5′-CCTAATTTTAGTCTT TCAAAACTGACATCTGTAATGCTTGTATGTATAA-3′ and 5′-TTATACATACAAGCATTACAGATGTCAGTTTTGAAAGACT AAA ATTAGG-3′, were used. The oligonucleotides were annealed and amplified by PCR. The resulting PCR product contained the two putative miR-181a binding sites, but with the seed sequences (5′-TGAATGT-3′) of miR-181a complementary sites, substituted with 5′-ACTGACA-3′. Cells were co-transfected with miR-181a together with psi-CHECK-PPFIA1-3′ UTR or psi-CHECK-PPFIA1-mut-3′ UTR and assayed for luciferase activity 24 h post-transfection using the Dual-Luciferase Reporter Assay System (E1910) (Promega). Firefly luciferase activity was normalized to that of Renilla luciferase for each sample.

MTT and Colony-Forming Assays

Cell viability was determined by MTT assays, as described previously.[12] The capacity for cell colony formation was determined by mixing cells with RPMI 1640 medium containing 0.9% methylcellulose solution, 20% FBS, 2 mM L-glutamine, and 5 μM 2-mercaptoethanol and seeding them in 24-well plates. Single cells were randomly and evenly distributed throughout the wells. Colonies were formed and counted 1 week later using an inverted microscope (Olympus, Japan). Colonies containing more than 50 cells were counted using the microscope. All analyses were performed in triplicate.

Cell Invasion Assays

The invasiveness of K562 cells was analyzed using invasion chambers (24 wells) coated with BD Matrigel matrix (8-mm pore size; BD

BioCoat, USA). A quantity of 600 μL of RPMI 1640 with 20% FBS, which served as chemoattractant, was added to the lower compartment of the invasion chamber. Cells (3×10^5) suspended in 100 μL of serum-free RPMI 1640 were seeded in the upper compartment of the invasion chambers and incubated for 8 h. After 8 h, cells at the top of the BD Matrigel matrix insert (apical side) were removed by gently rubbing the area with a cotton swab moistened with medium. Invaded cells on the lower side of the membrane were fixed with methanol for 30 min and stained with hematoxylin for 5–10 min. Photomicrographs of five random fields were taken (magnification ×100), and cells were counted to obtain the average number of cells that had invaded.

Stable Isotope Labeling and LC-MS/MS

RPMI 1640 medium was supplemented with 10% dialyzed fetal calf serum and all essential amino acids (Pierce) except L-lysine. The medium was then divided and supplemented with either $^{12}C_6$ L-lysine-2HCl or $^{13}C_6$ L-lysine-2HCl (Pierce) to produce light and heavy SILAC medium (provided by Guangzhou Fitgene Biotechnology), respectively. K562 cells were grown in parallel in both types of media and routinely passaged every 2–3 days (as appropriate) at 80%–90% confluence. After at least five cell doublings, the cells had achieved almost complete incorporation of heavy L-lysine. LC-MS/MS analysis was performed by Guangzhou Fitgene Biotechnology. The gel was cut into 48 slices from which proteins were digested and resulting peptides extracted and lyophilized before further analysis. Peptide powders were resuspended in solvent A (2% acetonitrile, 0.1% formic acid in water) and loaded onto a C18 reverse phase column (100 μm in diameter, 15 cm long, 3 μm resin from Michrom Bioresources, Auburn, CA, USA). Each peptide mixture was separated with a linear gradient of solvent B (5%–15%) for 15 min, followed by a gradient from 15%–35% for 85 min, and finally sustained at 90% for 20 min. Eluted peptides were injected directly on a linear trap quadrupole (LTQ)-Orbitrap XL (Thermo Fisher Scientific) through a nanoelectrospray ion source (Proxeon Biosystems) with a voltage of 1.85 kV and a transfer capillary temperature of 200°C. Data were acquired using Xcalibur software (Thermo Electron) in data-dependent mode. An accumulation of 106 ions was required to trigger a full MS scan, with a maximum accumulation time of 500 ms and a resolution of 60,000 (m/z 400), ranging from 400 to 2,000 Da. The six most intensive ions per MS scan were selected and fragmented by collision-induced dissociation (CID) in LTQ to perform the MS/MS scan with an accumulation of at least 5,000 ions and a maximum accumulation time of 100 ms. The normalized collision energy was 35%, activation Q was 0.25, and activation time was 30 ms; dynamic exclusion was enabled with a maximum retention period of 90 s and a relative mass window of 10 ppm. A lock mass (PCM, MW445.12) was introduced to improve the mass accuracy of survey scans.

IP, Co-immunoprecipitation, and Western Blotting

Different cell lines were lysed on ice in cell lysis buffer containing phenylmethylsulfonyl fluoride (PMSF) (Beyotime Biotechnology, Shanghai, China) for 30 min and then centrifuged at 12,000 × *g* for 30 min at 4°C. After centrifugation, clarified cell lysates were incubated with 15 μL of protein G plus/protein A-agarose and 1 μg of antibodies overnight at 4°C. Cell lysates, immunoprecipitates, and co-immunoprecipitates were resolved by SDS-PAGE and transferred to polyvinylidene fluoride (PVDF) membranes (Merck Millipore, Darmstadt, Germany). After washing, blots were incubated with primary antibody (see below), followed by horseradish peroxidase (HRP)-conjugated secondary antibody. Signals were visualized using enhanced chemiluminescence (ECL) (Merck Millipore, Darmstadt, Germany) and analyzed using a UVITEC Alliance 4.7 gel imaging system (Cambridge, UK).

Phospho-Specific Protein Microarray Analysis

Phospho-array detection was performed in collaboration with Wayen Biotechnology (Shanghai, China). After transfection with PPFIA1 siRNA or miR-181a mimic (72 h), treated K562 cells were collected for protein extraction. Protein samples (50 mg each) were labeled with biotin and hybridized to the Phosphorylation ProArray (Full Moon BioSystems, USA) using an Antibody Array Kit (Full Moon BioSystems, CA, USA) for the detection of 248 site-specific cancer signaling phospho-antibody profiles. Finally, fluorescence intensity was scanned with a GenePix 4000B (Axon Instruments, Houston, TX, USA) using GenePix Pro 6.0. Raw data were manipulated using Grubbs' method. The reduction phosphorylation ratio was calculated as follows: reduction phosphorylation ratio = phospho value/non-phosphorylated value.

Molecular Docking

The molecular structures of PPFIA1, PARP1, and ABL1 were obtained from PDB. Molecular docking was performed using Discovery Studio 4.5, following standard procedures as described in the software manual.

Protein Expression and Purification

Plasmids encoding hexa-histidine-tagged recombinant human ABL1 domain (isoform 2) were transformed into *Escherichia coli* [BL21 (DE3)]. After bacterial growth to an absorbance of 0.4–0.6 at 600 nm in Terrific Broth containing 30 mg/L kanamycin at 37°C, induction was carried out at 18°C using 0.5 mM isopropyl-β-D-thiogalactoside (IPTG), and growth continued at 18°C overnight. Bacteria were collected by centrifugation, and the obtained pellets were used immediately for the subsequent steps. Pellets were resuspended in lysis buffer (20 mM phosphate buffer [PB], 150 mM NaCl [pH 7.4]) containing protease inhibitor cocktail. Cell lysis was performed in an ultrasonic ice bath to generate crude protein samples. Cleaved protein samples were then diluted 5-fold with balance buffer (500 mM NaCl, 20 mM Tris [pH 8.0]) and incubated with Ni-agarose beads (CWBIO) to remove uncleaved protein and protease. Proteins were eluted from the beads with different concentrations of imidazole (20, 50, 200, and 500 mM) and the absorption peak detected. Subsequent samples were eluted using the imidazole concentration indicated by the absorption peak.

SPRi

SPRi assays were performed at 25°C in 1× PBS (pH 7.4) on a PlexArray HT (V3). The 3D UVC Chips were prepared to fix

berberine following standard Plexera protocol. For sequential binding assays, chips were pre-treated with berberine or mock-treated (rapamycin was used as a positive binding control) with a single injection (5 mL/min for 1 min) and then exposed to proteins. The retained resonance units (RUs) were recorded and triplicate values averaged.

Xenografted Mouse Model of BaF3-P210 and K562-Luciferase Cells and Immunohistochemistry

BALB/c nude and NSG mice (4–6 weeks old) were used for leukemogenesis experiments and maintained in a temperature- and humidity-controlled environment. A total of 1×10^6 BaF3-P210 cells were injected into sub-lethally irradiated (3 Gy) BALB/c mice, whereas K562-luciferase cells (Beijing Biocytogen) were injected into sub-lethally irradiated NSG mice intravenously via a tail vein. After injection of BaF3-P210 cells, BALB/c nude mice were intraperitoneally treated with 50 mg/kg olaparib in 0.2 mL of saline solution for 2 days. After injection of K562-luciferase cells, NSG mice were intravenously treated with 10 nmol PPFIA1-siRNA or NC-siRNA in 0.2 mL of saline solution for 2 days. Longitudinal follow-up was conducted to assess survival. Immunohistochemical analysis was performed on formalin-fixed, paraffin-embedded sections, as specified by Leica Biosystems. Anti-PPFIA1 and anti-PARP1 antibodies were used at a dilution of 1:50. Sections were processed and developed using a Leica Bond RX (Leica Biosystems). Images were obtained using a Panoramic 250 Flash Whole Slide Digital Scanner (Perkin Elmer). All animal experiments were conducted in accordance with a protocol approved by the Animal Care and Use Committee at Jinan University.

Statistical Analysis

The data are expressed as means ± SD of a minimum of three biological replicates. Statistical analysis was carried out by using Microsoft Excel and GraphPad Prism Software Version 6.01 (Systat Software, San Jose, CA, USA). Student's two-tailed unpaired t test was used to determine the significance, and p values <0.05 were considered statistically significant. The log rank test was used to determine the significant differences of the survival data, and p values <0.05 were considered statistically significant.

SUPPLEMENTAL INFORMATION

Supplemental Information includes four figures, five tables, Supplemental Materials and Methods, and six data files and can be found with this article online at https://doi.org/10.1016/j.omtn.2019.01.015.

AUTHOR CONTRIBUTIONS

J.F. conceived of and designed the experiments. C.G., Y. Liu, Z.Y., X.Z., and Y. Li performed the experiments. Y. Liu, Z.Y., J.Y., G.H., and C.G. analyzed the data. Z.Y. and Y. Liu contributed reagents, materials, and analytical tools. J.F., C.G., and Z.Y. wrote the paper.

CONFLICTS OF INTEREST

The authors declare no competing interests.

ACKNOWLEDGMENTS

This work was supported by grants from the National Natural Science Foundation of China (81170496), Research Project for Practice Development of National TCM Clinical Research Bases (JDZX2015119), Science and Technology Program of Guangdong Province (2014A020212729 and 2016A020226027), Science and Technology Program of Guangzhou City (201504281729050), and the Fundamental Research Funds for the Central Universities (17817021).

REFERENCES

1. Jabbour, E., and Kantarjian, H. (2018). Chronic myeloid leukemia: 2018 update on diagnosis, therapy and monitoring. Am. J. Hematol. *93*, 442–459.

2. Deininger, M.W., Goldman, J.M., and Melo, J.V. (2000). The molecular biology of chronic myeloid leukemia. Blood *96*, 3343–3356.

3. O'Brien, S.G., Guilhot, F., Larson, R.A., Gathmann, I., Baccarani, M., Cervantes, F., Cornelissen, J.J., Fischer, T., Hochhaus, A., Hughes, T., et al.; IRIS Investigators (2003). Imatinib compared with interferon and low-dose cytarabine for newly diagnosed chronic-phase chronic myeloid leukemia. N. Engl. J. Med. *348*, 994–1004.

4. Belloc, F., Airiau, K., Jeanneteau, M., Garcia, M., Guérin, E., Lippert, E., Moreau-Gaudry, F., and Mahon, F.X. (2009). The stem cell factor-c-KIT pathway must be inhibited to enable apoptosis induced by BCR-ABL inhibitors in chronic myelogenous leukemia cells. Leukemia *23*, 679–685.

5. Corbin, A.S., O'Hare, T., Gu, Z., Kraft, I.L., Eiring, A.M., Khorashad, J.S., Pomicter, A.D., Zhang, T.Y., Eide, C.A., Manley, P.W., et al. (2013). KIT signaling governs differential sensitivity of mature and primitive CML progenitors to tyrosine kinase inhibitors. Cancer Res. *73*, 5775–5786.

6. He, L., Thomson, J.M., Hemann, M.T., Hernando-Monge, E., Mu, D., Goodson, S., Powers, S., Cordon-Cardo, C., Lowe, S.W., Hannon, G.J., and Hammond, S.M. (2005). A microRNA polycistron as a potential human oncogene. Nature *435*, 828–833.

7. Chen, C.Z., Li, L., Lodish, H.F., and Bartel, D.P. (2004). MicroRNAs modulate hematopoietic lineage differentiation. Science *303*, 83–86.

8. Kozomara, A., and Griffiths-Jones, S. (2011). miRBase: integrating microRNA annotation and deep-sequencing data. Nucleic Acids Res. *39*, D152–D157.

9. Lin, S., Pan, L., Guo, S., Wu, J., Jin, L., Wang, J.C., and Wang, S. (2013). Prognostic role of microRNA-181a/b in hematological malignancies: a meta-analysis. PLoS ONE *8*, e59532.

10. Pekarsky, Y., Santanam, U., Cimmino, A., Palamarchuk, A., Efanov, A., Maximov, V., Volinia, S., Alder, H., Liu, C.G., Rassenti, L., et al. (2006). Tcl1 expression in chronic lymphocytic leukemia is regulated by miR-29 and miR-181. Cancer Res. *66*, 11590–11593.

11. Marcucci, G., Radmacher, M.D., Maharry, K., Mrózek, K., Ruppert, A.S., Paschka, P., Vukosavljevic, T., Whitman, S.P., Baldus, C.D., Langer, C., et al. (2008). MicroRNA expression in cytogenetically normal acute myeloid leukemia. N. Engl. J. Med. *358*, 1919–1928.

12. Fei, J., Li, Y., Zhu, X., and Luo, X. (2012). miR-181a post-transcriptionally downregulates oncogenic RalA and contributes to growth inhibition and apoptosis in chronic myelogenous leukemia (CML). PLoS ONE *7*, e32834.

13. Kwon, M.S., Kim, Y., Lee, S., Namkung, J., Yun, T., Yi, S.G., Han, S., Kang, M., Kim, S.W., Jang, J.Y., and Park, T. (2015). Integrative analysis of multi-omics data for identifying multi-markers for diagnosing pancreatic cancer. BMC Genomics *16* (*Suppl 9*), S4.

14. Rajewsky, N. (2006). microRNA target predictions in animals. Nat. Genet. *38* (*Suppl*), S8–S13.

15. Serra-Pagès, C., Medley, Q.G., Tang, M., Hart, A., and Streuli, M. (1998). Liprins, a family of LAR transmembrane protein-tyrosine phosphatase-interacting proteins. J. Biol. Chem. *273*, 15611–15620.

16. Astro, V., Asperti, C., Cangi, M.G., Doglioni, C., and de Curtis, I. (2011). Liprin-α1 regulates breast cancer cell invasion by affecting cell motility, invadopodia and extracellular matrix degradation. Oncogene *30*, 1841–1849.

17. Tan, K.D., Zhu, Y., Tan, H.K., Rajasegaran, V., Aggarwal, A., Wu, J., Wu, H.Y., Hwang, J., Lim, D.T., Soo, K.C., and Tan, P. (2008). Amplification and overexpression of PPFIA1, a putative 11q13 invasion suppressor gene, in head and neck squamous cell carcinoma. Genes Chromosomes Cancer *47*, 353–362.

18. Blessmann, M., Al-Dam, A., Hanken, H., Assaf, A.T., Riecke, B., Klatt, J., Simon, R., Sauter, G., Heiland, M., Kluwe, L., and Gröbe, A. (2013). Amplification of the PPFIA1 gene region on 11q13 in oral squamous cell carcinomas (OSCC). J. Craniomaxillofac. Surg. *41*, 845–849.

19. Shen, J.C., Unoki, M., Ythier, D., Duperray, A., Varticovski, L., Kumamoto, K., Pedeux, R., and Harris, C.C. (2007). Inhibitor of growth 4 suppresses cell spreading and cell migration by interacting with a novel binding partner, liprin alpha1. Cancer Res. *67*, 2552–2558.

20. Esposito, M.T., Zhao, L., Fung, T.K., Rane, J.K., Wilson, A., Martin, N., Gil, J., Leung, A.Y., Ashworth, A., and So, C.W. (2015). Synthetic lethal targeting of oncogenic transcription factors in acute leukemia by PARP inhibitors. Nat. Med. *21*, 1481–1490.

21. San José-Enériz, E., Román-Gómez, J., Jiménez-Velasco, A., Garate, L., Martin, V., Cordeu, L., Vilas-Zornoza, A., Rodríguez-Otero, P., Calasanz, M.J., Prósper, F., and Agirre, X. (2009). MicroRNA expression profiling in Imatinib-resistant Chronic Myeloid Leukemia patients without clinically significant ABL1-mutations. Mol. Cancer *8*, 69.

22. Souza, A.C., Azoubel, S., Queiroz, K.C., Peppelenbosch, M.P., and Ferreira, C.V. (2009). From immune response to cancer: a spot on the low molecular weight protein tyrosine phosphatase. Cell. Mol. Life Sci. *66*, 1140–1153.

23. Chiarugi, P., Taddei, M.L., Schiavone, N., Papucci, L., Giannoni, E., Fiaschi, T., Capaccioli, S., Raugei, G., and Ramponi, G. (2004). LMW-PTP is a positive regulator of tumor onset and growth. Oncogene *23*, 3905–3914.

24. Malentacchi, F., Marzocchini, R., Gelmini, S., Orlando, C., Serio, M., Ramponi, G., and Raugei, G. (2005). Up-regulated expression of low molecular weight protein tyrosine phosphatases in different human cancers. Biochem. Biophys. Res. Commun. *334*, 875–883.

25. Hou, J., Lin, L., Zhou, W., Wang, Z., Ding, G., Dong, Q., Qin, L., Wu, X., Zheng, Y., Yang, Y., et al. (2011). Identification of miRNomes in human liver and hepatocellular carcinoma reveals miR-199a/b-3p as therapeutic target for hepatocellular carcinoma. Cancer Cell *19*, 232–243.

26. Wolfrum, C., Shi, S., Jayaprakash, K.N., Jayaraman, M., Wang, G., Pandey, R.K., Rajeev, K.G., Nakayama, T., Charrise, K., Ndungo, E.M., et al. (2007). Mechanisms and optimization of in vivo delivery of lipophilic siRNAs. Nat. Biotechnol. *25*, 1149–1157.

27. Shim, M.S., and Kwon, Y.J. (2010). Efficient and targeted delivery of siRNA in vivo. FEBS J. *277*, 4814–4827.

28. Naughton, R., Quiney, C., Turner, S.D., and Cotter, T.G. (2009). Bcr-Abl-mediated redox regulation of the PI3K/AKT pathway. Leukemia *23*, 1432–1440.

29. Baum, K.J., and Ren, R. (2008). Effect of Ras inhibition in hematopoiesis and BCR/ABL leukemogenesis. J. Hematol. Oncol. *1*, 5.

30. Hoover, R.R., Gerlach, M.J., Koh, E.Y., and Daley, G.Q. (2001). Cooperative and redundant effects of STAT5 and Ras signaling in BCR/ABL transformed hematopoietic cells. Oncogene *20*, 5826–5835.

31. Sillaber, C., Gesbert, F., Frank, D.A., Sattler, M., and Griffin, J.D. (2000). STAT5 activation contributes to growth and viability in Bcr/Abl-transformed cells. Blood *95*, 2118–2125.

32. Steelman, L.S., Pohnert, S.C., Shelton, J.G., Franklin, R.A., Bertrand, F.E., and McCubrey, J.A. (2004). JAK/STAT, Raf/MEK/ERK, PI3K/Akt and BCR-ABL in cell cycle progression and leukemogenesis. Leukemia *18*, 189–218.

33. Kitamura, Y., and Hirotab, S. (2004). Kit as a human oncogenic tyrosine kinase. Cell. Mol. Life Sci. *61*, 2924–2931.

34. Yasuda, A., Sawai, H., Takahashi, H., Ochi, N., Matsuo, Y., Funahashi, H., Sato, M., Okada, Y., Takeyama, H., and Manabe, T. (2007). Stem cell factor/c-kit receptor signaling enhances the proliferation and invasion of colorectal cancer cells through the PI3K/Akt pathway. Dig. Dis. Sci. *52*, 2292–2300.

35. Schiewer, M.J., and Knudsen, K.E. (2014). Transcriptional roles of PARP1 in cancer. Mol. Cancer Res. *12*, 1069–1080.

36. Tobin, L.A., Robert, C., Rapoport, A.P., Gojo, I., Baer, M.R., Tomkinson, A.E., and Rassool, F.V. (2013). Targeting abnormal DNA double-strand break repair in tyrosine kinase inhibitor-resistant chronic myeloid leukemias. Oncogene *32*, 1784–1793.

37. Veuger, S.J., Hunter, J.E., and Durkacz, B.W. (2009). Ionizing radiation-induced NF-kappaB activation requires PARP-1 function to confer radioresistance. Oncogene *28*, 832–842.

38. Stilmann, M., Hinz, M., Arslan, S.C., Zimmer, A., Schreiber, V., and Scheidereit, C. (2009). A nuclear poly(ADP-ribose)-dependent signalosome confers DNA damage-induced IkappaB kinase activation. Mol. Cell *36*, 365–378.

39. Liu, S., Wu, L.C., Pang, J., Santhanam, R., Schwind, S., Wu, Y.Z., Hickey, C.J., Yu, J., Becker, H., Maharry, K., et al. (2010). Sp1/NFkappaB/HDAC/miR-29b regulatory network in KIT-driven myeloid leukemia. Cancer Cell *17*, 333–347.

OMTN, Volume *16*

Supplemental Information

Discovery of the Oncogenic Parp1, a Target of bcr-abl and a Potential Therapeutic, in mir-181a/PPFIA1 Signaling Pathway

Chunming Gu, Yanjun Liu, Zhao Yin, Juhua Yang, Guiping Huang, Xuejiao Zhu, Yumin Li, and Jia Fei

Supplemental Information

Supplemental Figures and Legends

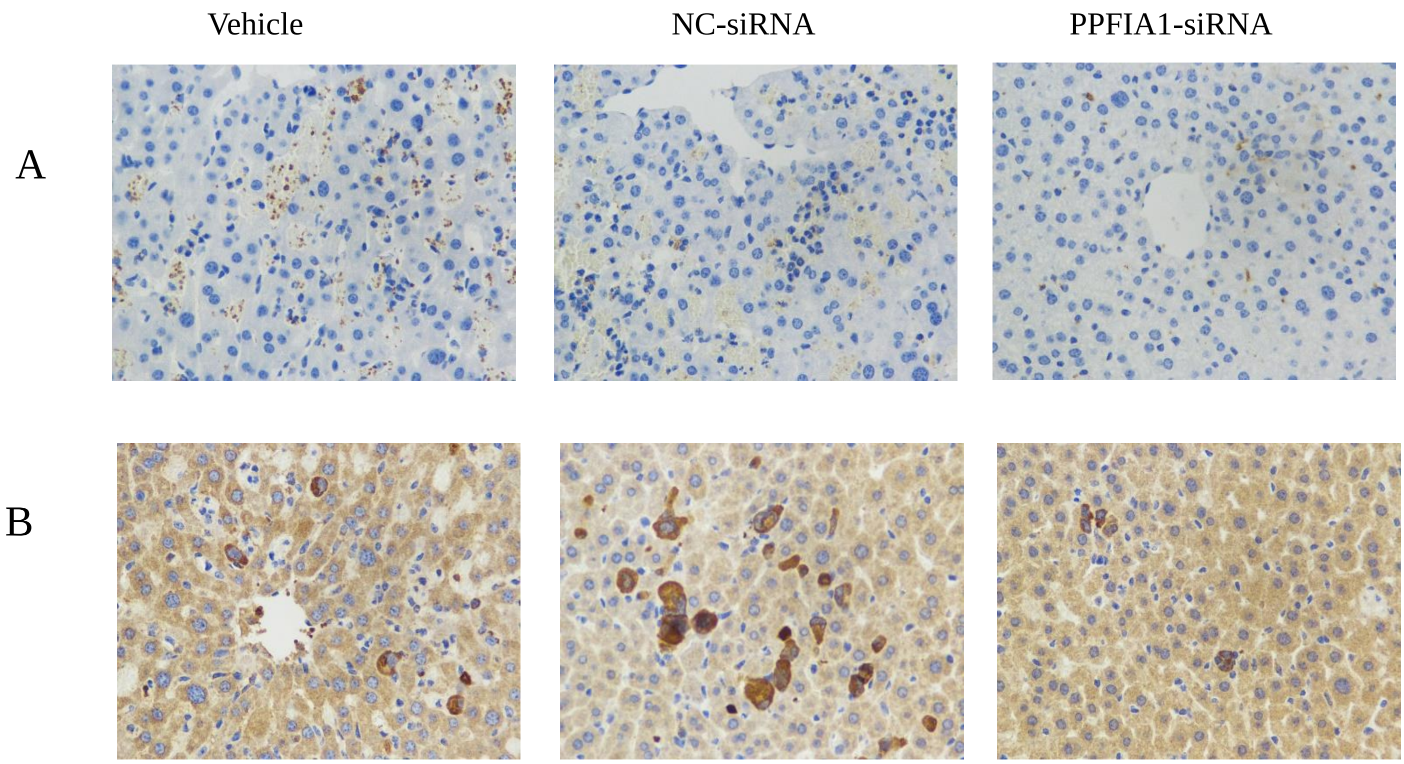

Figure S1. Immunohistochemical (IHC) analysis of liver samples from mice engrafted with K562-Luciferase cells untreated, NC-treated, and PPFIA1-siRNA-treated using anti-PPFIA1 (A) and anti-PARP1 (B) antibodies. Untreated and NC-treated mice were also used as controls.

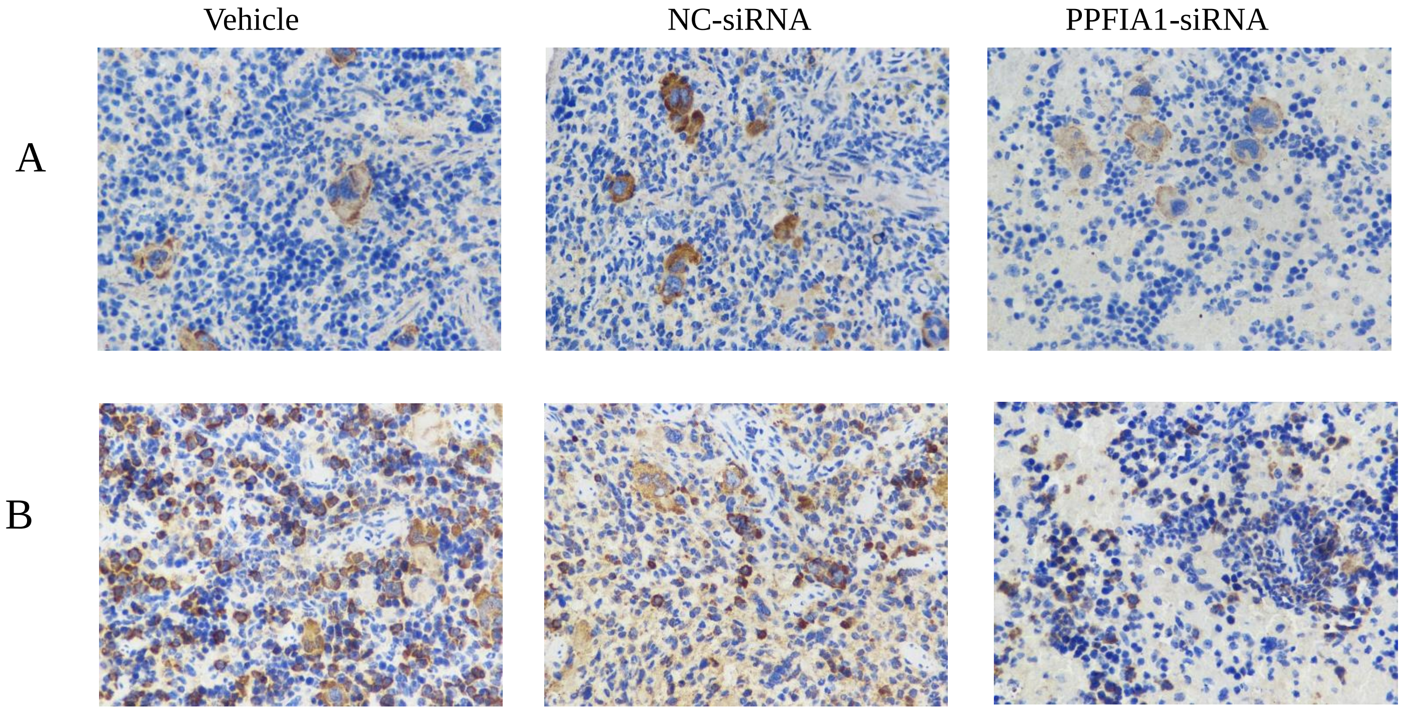

Figure S2. Immunohistochemical (IHC) analysis of spleens from mice engrafted with K562-Luciferase cells untreated, NC-treated, and PPFIA1-siRNA-treated using anti-PPFIA1 (A) and anti-PARP1 (B) antibodies. Untreated and NC-treated mice were also used as controls.

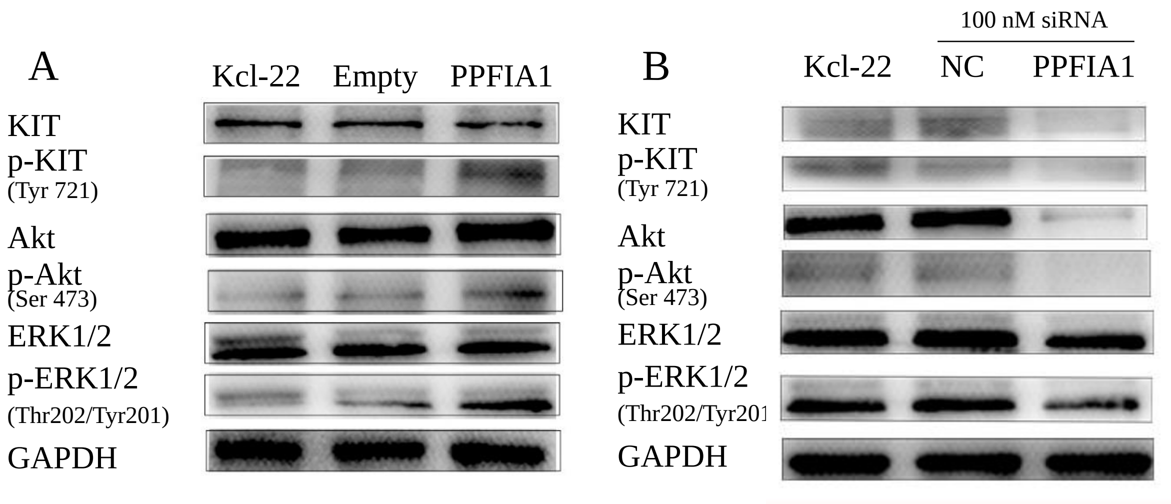

Figure S3. The effect of PPFIA1 on KIT signaling pathway.

(A) Overexpression of PPFIA1 increased the expression of phosphotylated KIT, Akt and ERK1/2 proteins in Kcl-22 cells. (B) Transfection of PPFIA1-siRNA for 48h attenuated the expression of phosphotylated KIT, Akt and ERK1/2 proteins in Kcl-22 cells.

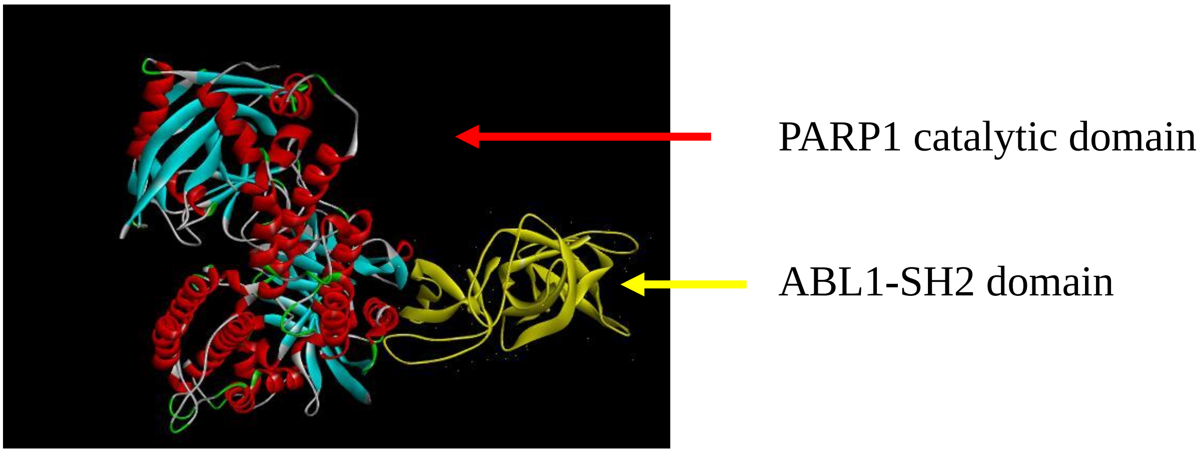

Figure S4. Docking of the PARP1 catalytic domain and the ABL1-SH2 domain.

Supplemental Tables

Table S1. Antibodies

REAGENT or RESOURCE	Supplier	IDENTIFIER
Antibodies		
Anti-GAPDH	Cell Signaling Technology	Cat# sc-74470
Anti-PPFIA1	Abcam	Cat# ab-96377
Anti-PPFIA1	Santa Cruz Biotechnology	Cat# sc-398030
Anti-C-KIT	Santa Cruz Biotechnology	Cat# sc-17806
Anti-p-C-KIT (Tyr 721)	Santa Cruz Biotechnology	Cat# sc-101659
Anti-PARP1	Santa Cruz Biotechnology	Cat# sc-74470
Anti-PARP	Cell Signaling Technology	Cat# 9532S
Anti-BCR	Abcam	Cat# ab-86173
Anti-c-Abl	Santa Cruz Biotechnology	Cat# sc-23
Anti-AKT	Cell Signaling Technology	Cat# 4691S
Anti-p-AKT (Ser 473)	Cell Signaling Technology	Cat# 4060S
Anti-ERK1/2	Cell Signaling Technology	Cat# 4695S
Anti-p-ERK1/2 (Thr 202/Thr 201)	Cell Signaling Technology	Cat# 9101S
Anti-rabbit IgG, HRP-linked	Cell Signaling Technology	Cat# 7074S
Anti-rabbit IgG, HRP-linked	Cell Signaling Technology	Cat# 7076S
Anti-P65	Santa Cruz Biotechnology	Cat# sc-514451
Anti-p-P65 (Ser 536)	Santa Cruz Biotechnology	Cat# sc-136548
Anti-FLAG	Sigma Aldrich	Cat# F1804

Table S2. Cell Lines

Cell lines	SOURCE
K562	Institute of Shanghai cell biology
293T	
KCL-22	Laboratory of Professor Markus Muschen
EM2	
Jurl-MK1	
B-luciferase K562	Beijing Biocytogen Co., Ltd.

Table S3. Experimental models

Experimental Model	SOURCE
Balb/c nu: female	Institute of laboratory animal science, JINAN University
C57BL/6	
B-NDG®(B-NSGTM) mice	Beijing Biocytogen Co., Ltd.

Table S4. Chemicals, recombinant proteins, and plasmids

Chemicals, Recombinant proteins, Plasmids	SOURCE	IDENTIFIER
Imatinib Mesylate (STI571)	Selleck.cn	S1026
Olaparib	Selleck.cn	S1060
PARP1 protein	Sino biological	11040-H08B-20
Anti-Flag Affinity Gel	Bimake	B23101
Psi-CHECK-2	Promega	C8021
PSI-CHECK-PPFIA1-3′UTR	This paper	N/A
PSI-CHECK-PPFIA1-MUT-3′UTR	This paper	N/A
LP-X0107-lv201-C0010	GeneCopoeia	N/A
LP-NEG-lv201-C0010	GeneCopoeia	N/A
LPP-X0107-Lv181-400	GeneCopoeia	N/A
LPP-NEG-Lv181-400	GeneCopoeia	N/A
EX-Z8307-Lv202	GeneCopoeia	N/A
EX-NEG-Lv202	GeneCopoeia	N/A
EX-Z4808-Lv201	GeneCopoeia	N/A
EX-NEG-Lv201	GeneCopoeia	N/A

Table S5. Oligonucleotides

Oligonucleotide	Sequence (5′ to 3′)	SOURCE
miR-181a mimic		Guangzhou RiboBio Co., Ltd.
sense	AACAUUCAACGCUGUCGGUGAGU	
antisense	UCACCGACAGCGUUGAAUGUUGU	
PPFIA1-siRNA		Guangzhou RiboBio Co., Ltd.
Sense	CCACAAAGCUCUGGAUGAAdTdT	
Antisense	dTdTGGUGUUUCGAGACCUACUU	
Scramble duplex, SCR		Guangzhou RiboBio Co., Ltd.
Sense	UUCUCCGAACGUGUCACGUTT	
Antisense	ACGUGACACGUUCGGAGAATT	
PPFIA1 primers		Sangon Biotech (Shanghai) Co., Ltd.
Forward	GCTTVAGTTAACCTTTTTCATGTATATCCA	
Reverse	TCGACAGTTAACATAAACGTAAAATCCTT	
GAPDH Primers		Sangon Biotech (Shanghai) Co., Ltd.
Forward	CAACGGATTTGGTCGTATT	
Reverse	CACAGTCTTCTGGGTGGC	
KIT Primers		Sangon Biotech (Shanghai) Co., Ltd.
Forward	CAATGGCACGGTTGAATGTA	
Reverse	AAGGAGTGAACAGGGTGTGG	
PARP1 Primers		Sangon Biotech (Shanghai) Co., Ltd.
Forward	CCGCATACTCCATCCTCAGT	
Reverse	GCTTCTTCATCCCAAAGTCG	
P65 Primers		Sangon Biotech (Shanghai) Co., Ltd.

Forward	GGAGCACAGATACCACCAAGA
Reverse	CGGCAGTCCTTTCCTACAAG

Supplemental Methods

Immunohistochemical staining and image analysis

The slides were deparaffinised and rehydrated following the manufacturer's instructions. Endogenous peroxidase activity was abolished by 3% H_2O_2, antigen retrieval was conducted in citrate buffer. Slices were incubated with a primary antibody (anti-PARP1 or PPFIA1 at 1:50 dilution) at 4 ℃ overnight, followed by incubation with a HRP-labeled secondary antibody for 30 min. Images were obtained by a Leica DMRI microscope installed on a Lexica DC500 camera (Leica).
